# Rethinking Chlorine: Essential Chemical or Replaceable Risk?

**DOI:** 10.1002/cssc.202402697

**Published:** 2025-05-06

**Authors:** Johannes Schwan, Merlin Kleoff, Gesa H. Dreyhsig, Patrick Voßnacker, Traute Fiedler, Marian Rosental, Sebastian Riedel

**Affiliations:** ^1^ Umweltbundesamt Section III 2.1, Wörlitzer Platz 1 06844 Dessau‐Roßlau Germany; ^2^ Fachbereich für Biologie, Chemie, Pharmazie Institut für Chemie und Biochemie – Anorganische Chemie Freie Universität Berlin Fabeckstr. 34/36 14195 Berlin Germany; ^3^ Institut für Energie‐ und Umweltforschung Heidelberg gGmbH Wilckensstraße 3 69120 Heidelberg Germany; ^4^ Center for Sustainable Resources – CSR Berlin Freie Universität Berlin Fabeckstrasse 34/36 14195 Berlin Germany

**Keywords:** chlorine, environmental chemistries, industrial chemistries, ionic liquids, sustainable chemistries

## Abstract

This review critically examines the dual nature of chlorine as both an indispensable base chemical and a potential risk. Chlorine and its byproduct hydrogen chloride play essential roles in the production of pharmaceuticals, plastics, agrochemicals, and disinfectants. However, their inherent toxicity, risks of handling, and environmental impacts necessitate a reassessment of their use and sustainability. The review explores emerging and established chlorine‐free technologies, such as the hydrogen peroxide to propylene oxide process and phosgene‐free routes for polycarbonate production, evaluating their potential to reduce reliance on chlorine. For applications where chlorine remains indispensable, innovations such as trichloride‐ and bichloride‐based ionic liquids provide safer storage and handling options for chlorine and hydrogen chloride, respectively. These ionic liquids not only enhance safety but also support renewable energy integration through their potential as indirect energy storage solutions. While chlorine is unlikely to be fully replaced in the near future, ongoing innovations in chlorine‐free processes and safer technologies may redefine its industrial use, contributing to a more sustainable and secure chemical industry.

## Graphical abstract

Chlorine is essential for the production of plastics and pharmaceuticals but poses significant safety and environmental risks. Herein, processes are presented that substitute chlorine or reduce its demand. Where indispensable, trichloride‐ and bichloride‐based ionic liquids could enhance safety and streamline the integration of renewable energy, contributing to a more sustainable and safe chemical industry.

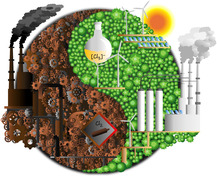

## 1 Introduction

Chlorine (Cl) is the 17th element in the periodic table and belongs to the group of halogens. As it appears at room temperature (RT) as a yellow–greenish gas, it was named after the ancient Greek χλωρóϛ (chloros, pale green). Elemental chlorine occurs in the form of the dichlorine molecule (Cl_2_). As halogens in general, chlorine is very reactive, which is why it is one of the most popular chemical building blocks in the chemical industry, but can also cause major problems if released into the environment.^[^ ^1^, ^2^, ^3^, ^4^, ^5^ ^]^

In contrast to elemental chlorine, in nature chlorine can mainly be found in the form of chloride (Cl^−^) salts. These salts are present in significant quantities in rocks and (sea) water. It is essential for almost all biological organisms, which in turn incorporate it into countless natural substances.^[^ ^1^, ^2^, ^3^, ^4^, ^5^ ^]^

### 1.1 The Controversial Role of Chlorine

Probably only few chemicals have had such an impact on the development of the civilized world as chlorine—being the feedstock for valuable materials and public health achievements, but also causing severe environmental problems.^[^ ^1^, ^2^, ^3^, ^4^, ^5^ ^]^

In public perception, chlorine is seen primarily as a dangerous chemical. Chlorine itself has been used along with other chlorinated compounds including phosgene (COCl_2_) and mustard gas (bis(2‐chloroethyl) sulfide) as chemical warfare agent.^[^ ^6^, ^7^, ^8^ ^]^

The chlorine industry is still associated with environmental disasters of the past such as the Seveso accident in 1976 where unknown amounts of the toxic dioxin 2,3,7,8‐tetrachlordibenzo‐*p*‐dioxin (TCDD) have been released into the environment.^[^ ^9^ ^]^ While the area around the industrial plant was heavily contaminated for years, 193 people were directly injured suffering from chloracne (88% of whom were children under the age of 15).^[^ ^10^ ^]^

The ambivalence in the use of chlorinated substances can be illustrated by their utilization as agrochemicals. These chemicals are in general more stable than their nonchlorinated counterparts.^[^ ^11^ ^]^ This offers the advantage that they have to be used less frequently while maintaining a reliable crop protection—on the other hand, the regular use of chlorinated agrochemicals can lead to environmental problems due to their persistent nature. A prominent, yet controversial example that showcases the advantages but also disadvantages of chlorinated insecticides is dichlorodiphenyltrichloroethane (DDT). The insecticidal properties of this chlorinated compound have been discovered by the Swiss chemist Paul Hermann Müller, for which he was awarded with the Nobel Prize for medicine in 1948.^[^ ^12^ ^]^ After its launch, DDT was heavily used in agricultural pest control, replacing the less effective and more toxic lead arsenate.^[^ ^12^, ^13^, ^14^ ^]^ Most importantly, DDT was (and, to a small extend, still is) used for indoor residual spraying to fight malaria. This application of DDT reduced the cases of malaria in India from 100 million in 1933 to 0.15 million in 1966.^[^ ^12^ ^]^ In total, it is estimated that the use of DDT to fight malaria saved 5 million human lives and prevented 100 million infections in the first ten years after its launch.^[^ ^15^ ^]^

On the other hand, the widespread use of DDT caused environmental problems as it is persistent in nature and accumulates in the food chain, which contributed, for instance, to a population decline of several bird species in North America and Europe.^[^ ^12^, ^16^ ^]^ Consequently, starting in the 1970s, the agricultural application of DDT was more restricted or prohibited.^[^ ^17^ ^]^ However, DDT remains in use today for malaria control in some countries, where its effectiveness continues to make it a valuable tool in the fight against this life‐threatening disease. A complete abandonment of DDT will probably require the further development of environmentally friendly and economically viable insecticides.^[^ ^18^ ^]^

Chlorine itself is successfully used for the purification of drinking water for over one century.^[^ ^19^ ^]^ The availability of clean drinking water is of critical importance for public health, given the prevalence of waterborne diseases such as cholera, typhoid fever, and hepatitis A and E.^[^ ^20^ ^]^ According to the World Health Organization (WHO), 1.7 billion people globally still lack access to safe drinking water, resulting in ≈500 000 deaths annually.^[^ ^21^ ^]^ As a result, the United Nations has prioritized “ensuring availability and sustainable management of water and sanitation for all” as one of its 17 Sustainable Development Goals.^[^ ^22^ ^]^ In the late 19th century, chlorine‐based water treatment methods were introduced in the United States and Europe, leading to a significant reduction in the incidence of waterborne diseases in these regions. Today, chlorination or the use of chlorinated compounds remains a common disinfection practice in developed nations.^[^ ^19^ ^]^

Although there are concerns regarding the formation of chlorine‐containing disinfection byproducts (DBPs),^[^ ^20^, ^23^ ^]^ the WHO stated that the use of chlorine in water treatment has had substantial public health benefits, which outweigh the potential risks associated with DBPs.^[^ ^24^ ^]^

### 1.2 The Importance of Chlorine for the Chemical Industry

Besides these prominent examples, chlorine has influenced almost every branch of the chemical industry—and modern life. It is estimated that more than 50% of all industrial chemicals and polymers rely on chlorine chemistry, including important polymers such as polyvinyl chloride (PVC), polyurethane (PU), polycarbonate (PC), and silicones.^[^ ^1^, ^25^ ^]^

Chlorine chemistry plays a crucial role in the production of silicon, which is necessary for semiconductors and solar cells, and for inorganic materials such as titanium dioxide (TiO_2_) and phosphorus trichloride (PCl_3_).^[^ ^26^ ^]^

**Figure 1:**
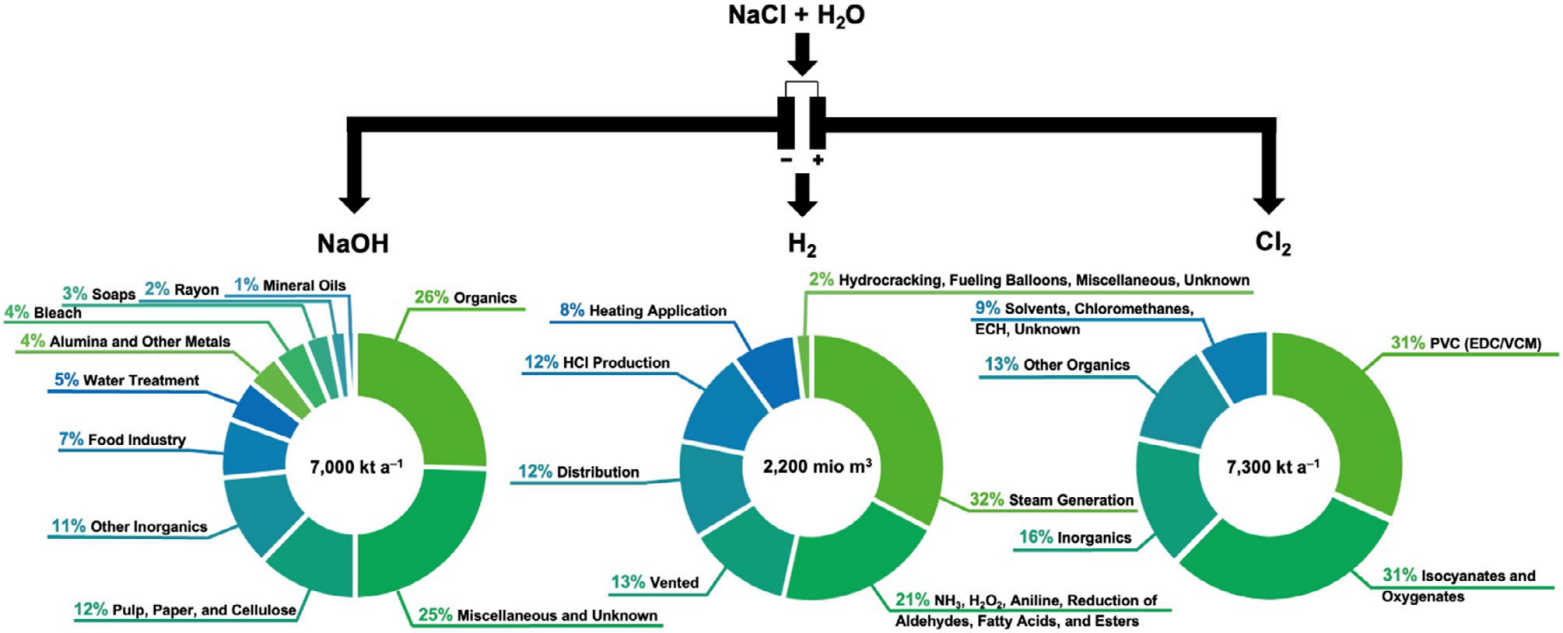
The primary products of the chloralkali electrolysis (NaOH, H_2_, and Cl_2_) and their applications in Europe.^[^ ^28^ ^]^

In addition, more than 90% of all pharmaceuticals and 86% of all agrochemicals contain or are synthesized using chlorine or chlorine‐derived chemicals.^[^ ^2^ ^]^ Among the chlorine‐containing pharmaceuticals are drugs such as Diazepam (anxiolytic), Ketamin (anesthetic), and Chlorhexidin (antiseptic) that are included in the list of the World Health Organization's List of Essential Medicines.^[^ ^27^ ^]^ In Europe, 7.3 million tons of chlorine were produced in 2023, which have been primarily used for the manufacturing of polymers (PVC: 32.0%; PU and PC: combined 30.7%. **Figure** **1**).^[^ ^28^ ^]^

To meet the global chlorine demand, in 2023, ≈100 million tons chlorine have been produced worldwide, primarily by chloralkali electrolysis. This process provides besides chlorine also hydrogen and caustic soda (NaOH). While the provided hydrogen is mainly used for steam generation, as well as the production of ammonia, hydrogen peroxide, and pure hydrogen chloride, caustic soda is a base chemical with countless applications ranging from the pulp and paper industry to the production of aluminum and soaps.^[^ ^28^ ^]^ However, the chloralkali electrolysis is highly energy intensive consuming about ≈2.6 MWh t^−1^ of chlorine, making the energy costs responsible for 67–77% of the total production costs of chlorine in Europe.^[^ ^28^, ^29^ ^]^ As a result, in Germany, the largest chlorine producer in Europe, the chloralkali electrolysis consumed ≈1.7% of the German electricity in 2022 (for details, see the Supporting Information, Section 1). The high‐energy demand of the chloralkali electrolysis accounts also for significant CO_2_ emissions of the chlorine industry which are similar to those of the iron and steel industry.^[^ ^2^ ^]^ On the other hand, given the high reactivity of chlorine, all large industrial processes utilizing elemental chlorine are exothermic, generating process heat that is ideally used for other processes.

Notably, the chloralkali electrolysis is not only the primary source of chlorine, but also of 99.5% of the globally produced caustic soda (in 2023: ≈82 Mt globally).^[^ ^30^, ^31^, ^32^, ^33^ ^]^ The remaining 0.5% are related to other processes, such as the causticization of sodium carbonate (Na_2_CO_3_) using calcium oxide (CaO) in the presence of water to produce sodium hydroxide.^[^ ^31^ ^]^ However, this process requires the lime kiln of CaCO_3_ to provide CaO, which suffers from an inherently high carbon footprint.^[^ ^32^ ^]^ Thus, the production of chlorine and of caustic soda is highly interconnected and to meet the global need of caustic soda, also chlorine has to be produced and used, as chlorine cannot be released into the environment.^[^ ^34^ ^]^ To avoid the global use of chlorine, it will probably be necessary to find an industrially useful alternative to the established chloralkali electrolysis for the production of caustic soda as well as alternative processes avoiding the use of caustic soda.^[^ ^31^ ^]^

The high industrial value of chlorine can mainly be attributed to two facts.

1 Chlorine is very abundant and cheap. Its production relies on almost unlimited available sodium chloride and water as starting materials. Despite the high‐energy demand of the chloralkali electrolysis, chlorine is one of the cheapest base chemicals with a price of ≈200 € t^−1^ in Europe in 2019 (for details, see the Supporting Information, Section 2).
2 Chlorine is a strong oxidant, able to react with countless organic and inorganic materials. In organic compounds, chlorine forms C—Cl bonds with a moderate bond strength of 338 kJ mol^−1^ making them comparably stable but still reactive enough to serve as a leaving group in substitution and elimination reactions.^[^ ^2^, ^35^ ^]^

Notably, not all materials that are produced via chlorine chemistry do contain chlorine in the final product. For example, polyurethane (PU) is a chlorine‐free polymer, but is produced using phosgene (COCl_2_) which is derived from chlorine (Cl_2_) and carbon monoxide (CO). During the manufacturing of chlorine‐free materials, large amounts of hydrogen chloride or chloride salts (mainly NaCl) are released. Hydrogen chloride is partially recycled (e.g., by electrolysis or the Deacon process), partially used for other processes, or, if a utilization is not possible, typically neutralized with NaOH forming NaCl solutions.^[^ ^36^ ^]^ These chloride‐containing wastes can harm the environment when discharged into rivers in larger quantities as it increases the chloride concentration. High salinity in combination with other factors such as an increased temperature, reduced water flow, and sunlight has recently led to a dramatic growth of toxic algae in the Oder River in Germany, which caused a mass fish die‐off in 2022.^[^ ^37^, ^38^ ^]^ Therefore, the reduction of chloride wastes or their recycling is crucial to prevent harm from watersheds and ecosystems.^[^ ^39^ ^]^

Even more problematic than chloride‐containing waste streams are many chlorinated organic compounds such as DDT (see above), hexachlorocyclohexane isomers, and polychlorinated biphenyls.^[^ ^40^ ^]^ One important parameter to monitor organic chlorine pollution is the adsorbable organic halogens (AOX) parameter, indicating the total amount of water‐soluble chlorinated, brominated, and iodinated organic halogen compounds.^[^ ^41^, ^42^ ^]^ Many of these compounds are toxic, mutagenic, or carcinogenic and possess long half lives in the environment, high lipophilicity, and have the ability to bioaccumulate in food chains.^[^ ^41^ ^]^ While more than 5000 organic halogen compounds are naturally produced by living organisms, volcanoes, or forest fires, the majority of anthropogenically produced AOX are generated during industrial processes.^[^ ^43^ ^]^

Related to the AOX parameter is the total organic carbon (TOC) value which is a measure of the total amount of carbon bound in organic compounds present in a sample, typically water, soil, or air from both natural and anthropogenic sources. The TOC is typically used as an indicator of the overall level of organic pollution particularly in water.^[^ ^44^ ^]^ High TOC levels can indicate contamination by organic pollutants, which may affect aquatic life or human health.^[^ ^45^ ^]^ A reduction of chlorination processes, where feasible, could contribute to lower AOX and TOC values, helping to mitigate potential environmental impacts on water bodies.^[^ ^3^, ^41^ ^]^

### 1.3 The Safety Challenges of Chlorine and Hydrogen Chloride

The reactivity of chlorine makes it a versatile chemical building block in industry—but also dangerous to human life when released accidentally in the environment.^[^ ^4^, ^5^ ^]^ Chlorine is usually stored by pressure liquefaction building up a vapor pressure of 6.8 bar at RT and requires the use of resistant materials to deal with its high corrosiveness.^[^ ^4^ ^]^ Accordingly, and due to its high toxicity, the transport and storage of chlorine pose significant safety risks.^[^ ^46^, ^47^, ^48^ ^]^ Although most chlorine is consumed at the production site, it is occasionally transported in pressurized tanks, leading to potential risks, as it has been witnessed in 2022 by an accident in Aqaba, Jordan, where 13 people died and more than 250 were injured by the rupture of a chlorine vessel.^[^ ^49^ ^]^ Such incidents, though rare, highlight the importance of stringent safety measures in chlorine handling.

For safety reasons, the storage of chlorine is limited with most production facilities maintaining only relative small buffer stocks to accommodate supply fluctuations, whereas long‐term storage of chlorine is avoided due to the risks associated with its high toxicity, corrosiveness, and the formation of heavy gas clouds near the ground when leaked.^[^ ^4^, ^50^, ^51^ ^]^ Cl_2_ is a strong and very reactive oxidizer and although it is inflammable itself, it can easily form combustible and explosive mixtures with a variety of chemical gases like hydrogen, hydrocarbons, and ammonia.^[^ ^5^, ^52^ ^]^ However, many industrial processes depend on mixing these gases with each other, so it must be possible to perform the reactions under safe and controlled conditions using suitable materials, facilities, and adapted safety equipment.^[^ ^4^, ^53^ ^]^

In addition to chlorine itself, other chlorine‐derived compounds, such as hydrogen chloride (HCl), are widely used in industrial processes and share similar safety concerns. Hydrogen chloride is another highly toxic, corrosive but colorless gas at RT. In analogy to chlorine, it is usually stored by pressure liquefaction building up a large vapor pressure of 42.6 bar. Thus, it is mainly used directly on‐site to produce other chemicals.^[^ ^54^, ^55^ ^]^ In addition to being produced as a byproduct of the chlorine industry, hydrogen chloride is also produced explicitly to obtain a very pure product, which is then used, for example, for the semiconductor industry.^[^ ^55^ ^]^ One way to do this is the reaction directly from the elements, chlorine and hydrogen.^[^ ^55^ ^]^ In this case, Cl_2_ and H_2_ are mixed in a combustion chamber, where the mixture is ignited to enable the controlled combustion of chlorine with hydrogen in a very hot flame reaching temperatures >2000 °C.^[^ ^55^ ^]^ While this reaction is performed under controlled conditions, mixtures of Cl_2_ and H_2_ must be treated with specific care due to their high explosive potential over a wide range with a lower explosion limit of 4 vol.‐% and an upper explosion limit of 89 vol.‐%.^[^ ^56^ ^]^ The reaction is highly exothermic with a standard enthalpy of reaction of Δ_R_ *H*° = –184 kJ mol^−1^ and can be induced by heat or even light.^[^ ^55^ ^]^ To deal with these extreme reaction conditions and the high corrosiveness and toxicity of HCl and Cl_2_, materials with a great resilience must be used and the staff must be specially trained.^[^ ^4^, ^52^, ^55^ ^]^ However, the use of the right materials does not prevent the abrasion of pipes, reactors, and controlling systems and thus, a high flexibility of all processes containing Cl_2_ and HCl is needed to enable a constant control and potential replacement of affected parts.^[^ ^57^, ^58^ ^]^ However, the unintentional release of hydrogen chloride or chlorine comes along with high risks for human life and the environment and cannot be totally excluded even using proper equipment and adhering to the required safety standards.^[^ ^52^, ^57^ ^]^ Incidents of chlorine gas release in pool facilities underscore the critical importance of strict safety protocols where chlorine‐based disinfection is used. Regular training, equipment maintenance, and adherence to safety standards are essential to minimize risks to personnel and the public.^[^ ^59^, ^60^ ^]^

### 1.4 Scope of this Review

In view of the disadvantages of chlorine and related compounds in terms of sustainability and safety, the necessity of minimizing the utilization of chlorine has been underscored. In the context of sustainable development, chlorine‐free alternatives are being increasingly explored as a means to reduce environmental burdens and health risks associated with chlorine‐based processes.^[^ ^2^, ^3^ ^]^

On the other hand, chlorine is deeply embedded in the chemical industry, particularly in pharmaceuticals, agrochemicals, and polymer manufacturing. Substituting established chemical processes by new ones is technically challenging and economically complex, particularly given the low cost and high availability of chlorine.^[^ ^1^, ^3^ ^]^ As described earlier, the production of caustic soda by chloralkali electrolysis is tightly linked with chlorine production, meaning that as long as caustic soda is produced, chlorine is also generated and must be utilized, as it cannot simply be released into the environment.

This review will examine alternative processes that avoid or minimize the use of chlorine and evaluate their technological readiness, waste reduction potential, and practical application within industrial settings. Chlorine‐free methodologies, which range from novel synthetic routes to ecofriendly catalysts, are analyzed based on their ability to replace traditional chlorine‐based reactions while maintaining or enhancing efficiency and safety. Their environmental footprint, particularly in terms of waste, emissions, and energy consumption, is compared to that of chlorine‐based processes to provide a picture of their sustainability and viability. In addition, contemporary technologies based on ionic liquids (ILs) to tame the inherent hazards of chlorine or hydrogen chloride will be reviewed.

This review is partially based on a recent report from the German Environment Agency (Umweltbundesamt) concerning environmentally friendlier alternative production processes.^[^ ^61^ ^]^ Therefore, the provided data regarding production scales and emission profiles is mainly limited to the German market, while the insights offered have global relevance, as the chlorine‐free alternative processes reviewed here are essentially applicable and beneficial to the chlorine chemistry worldwide.

## 2 Alternative Processes for the Production of Relevant Chemicals without Chlorine

A recent report of the German Environment Agency on reduction potentials and alternative production methods in the chemical sector identified the most chlorine demanding processes in Germany and quantified their absolute chlorine consumption for 2017.^[^ ^61^ ^]^

The total chlorine demand was calculated to be ≈3.8 Mt for the production of chlorine‐derived chemicals in Germany based on various production volumes, process requirements, and estimates. This corresponds to around 93% of the total amount of chlorine produced in Germany in 2017 (4.1 Mt).^[^ ^62^ ^]^

For a detailed analysis of alternative processes that avoid the use of chlorine, chemicals were selected that have a high absolute chlorine demand during production, but do not contain chlorine themselves; or contain chlorine, but are completely processed into chlorine‐free products (e.g., epichlorohydrin (ECH) to epoxy resins). These include

1 propylene oxide (PO) (1.1 Mt Cl_2_/27%);
2 phosgene for polyurethanes (820 kt Cl_2_/20%);
3 ECH (327 kt Cl_2_/8%);
4 chlorinated methane derivatives (CMDs) (222 kt Cl_2_/5%) for solvents and tetrafluoroethylene (TFE) synthesis; and
5 phosgene for polycarbonates (122 kt Cl_2_/3%).

Although the production of PVC requires the second highest amount of chlorine (907 kt/22%) after PO, it is not included in Section 2 since the final product (PVC) itself contains chlorine. Furthermore, the optimization potential in terms of chlorine consumption is considered to be very low due to efficient process control of chlorination and oxychlorination as well as high recovery rates of hydrogen chloride released during vinyl chloride (VCM) manufacturing.^[^ ^63^ ^]^

Due to the lack of industrially feasible alternatives, also the production of silicones and titanium dioxide is not covered in this review. However, further details on new synthetic approaches to these chlorine intensive materials can be found in the report of the German Environment Agency.^[^ ^61^ ^]^

### 2.1 Propylene Oxide (PO)

PO is mainly required for the production of polymers such as polyurethanes and polyesters but also for solvents like propylene glycol ethers.^[^ ^64^ ^]^ An estimated amount of 2,860 kt of PO was produced in the EU in 2017.^[^ ^65^ ^]^ Although PO itself does not contain chlorine, its production consumed 1.1 Mt of chlorine in Germany in 2017.^[^ ^61^ ^]^ PO producers in Germany are Dow Chemical in Stade (capacity 630 kt a^−1^), INEOS Oxide in Cologne (210 kt a^−1^), and BASF in Ludwigshafen (120 kt a^−1^).^[^ ^65^ ^]^

In the context of this review, the most relevant industrial PO processes are briefly described and evaluated for their environmental performance. For a more detailed description of different industrial PO syntheses, see Baer et al.^[^ ^64^ ^]^

#### 2.1.1 Chlorohydrin Process

**Scheme 1:**
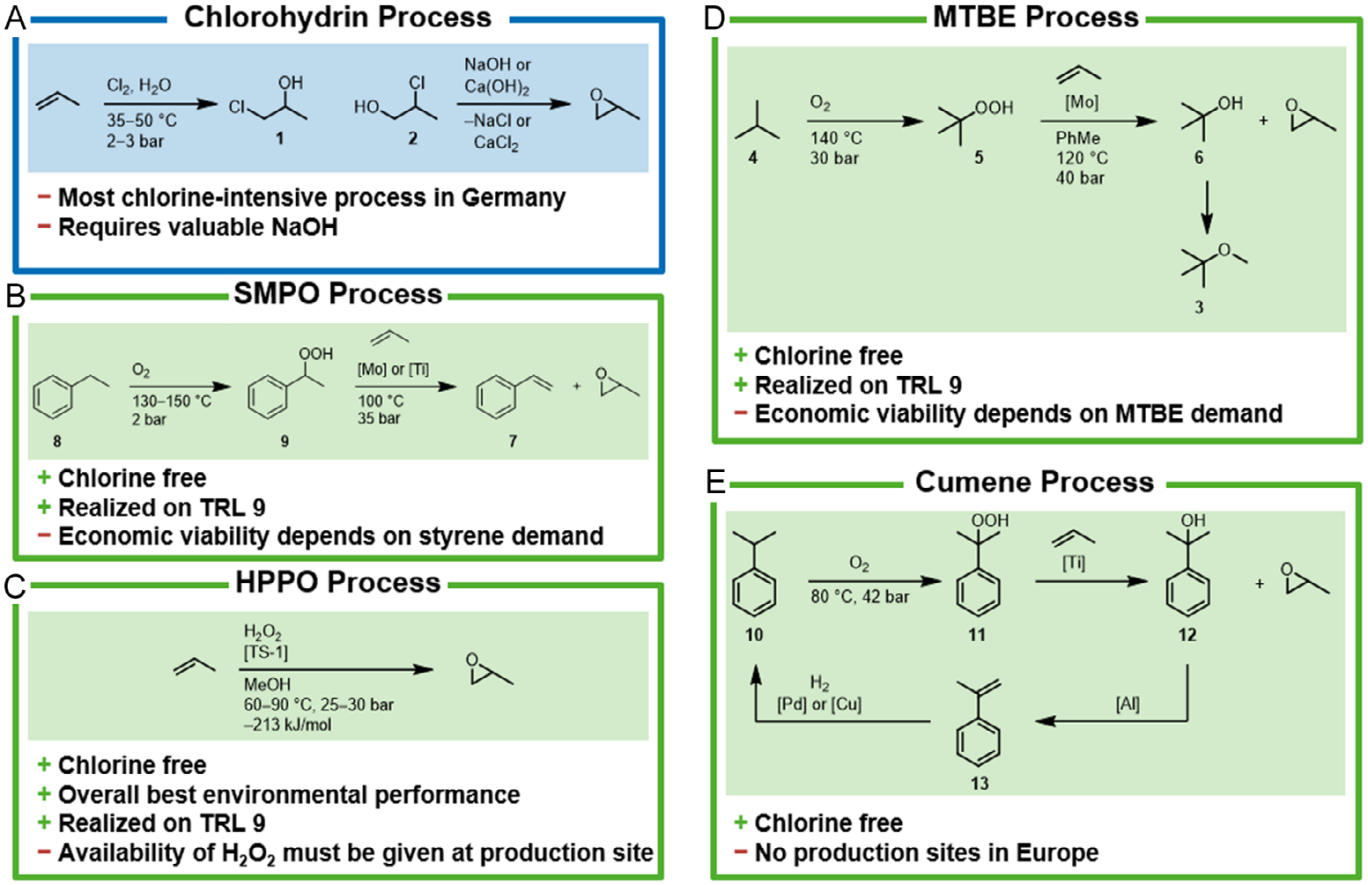
A) Conventional PO process via chlorohydrin. B–E) Chlorine‐free alternatives. TRL = technology readiness level (measuring the maturity of a technology from 1 (basic research) to 9 (proven in operational environment)).

The capacity of the chlorohydrin process accounts for a total of 30% of European PO production (as in 2022).^[^ ^66^ ^]^ In Germany, PO is produced exclusively using the chlorohydrin process. Here, hypochlorous acid is generated in situ from chlorine and water at 35–50 °C and 2–3 bar, which reacts with excess propylene to form both propylene chlorohydrin isomers **1** and **2** (**Scheme** **1**A).^[^ ^64^ ^]^ In the next step, both isomers are dehydrochlorinated at 25 °C in the presence of NaOH or Ca(OH)_2_. PO is separated from other low‐boiling substances by distillation and can be obtained with a selectivity of 90%. In addition, other chlorinated byproducts of minor economic importance are formed (e.g., dichloropropane).^[^ ^67^, ^68^ ^]^ The chlorine requirement is ≈1.35 t Cl_2_/t PO.^[^ ^66^ ^]^

#### 2.1.2 MTBE Process

In addition to the chlorohydrin process, coproduct processes have also been established whose economic viability is based on the sale of a coproduct in addition to PO. One of these processes is the MTBE process in which methyl *tert*‐butyl ether (MTBE, **3**) is formed alongside PO (Scheme 1B). Isobutane (**4**) is first converted with the aid of molecular oxygen at up to 140 °C and 30 bar without a catalyst to form *tert*‐butyl hydroperoxide (**5**), which partially decomposes to *tert*‐butyl alcohol (**6**). Small quantities of ketones and aldehydes are also formed. This crude mixture is dissolved in toluene, a molybdenum catalyst is added and mixed with propylene. The reaction mixture is passed through several reactor stages in which the temperature is gradually increased from 110 to 120 °C at a pressure of 40 bar with an approximately tenfold excess of propylene in the mixture affording PO and *tert‐*butyl alcohol (**6**).^[^ ^64^ ^]^ After the reaction, crude PO is separated by distillation, which still contains other hydrocarbons, carbonyls, and ethylene oxide, which are removed by further purification steps. The *tert*‐butyl alcohol separated in the first distillation step is converted into MTBE by reaction with methanol. For each kg of PO produced, 2.98 kg of *tert*‐butyl alcohol (**6**) are formed.^[^ ^64^, ^69^ ^]^

#### 2.1.3 SMPO Process

Another technology based on a coproduct is the styrene monomer PO (SMPO) process, where styrene (**7**) is formed alongside PO (Scheme 1C).^[^ ^64^, ^70^ ^]^ Here, ethylbenzene (**8**) (prepared from benzene and ethylene) is first reacted with air at 130–150 °C and 2 bar to form ethylbenzene hydroperoxide (**9**), which is obtained as a solution of only 10–14 wt% in ethylbenzene due to safety reasons.^[^ ^64^, ^71^ ^]^ The organic hydroperoxide is used as an oxygen carrier to oxidize propylene and to produce styrene (**7**) at 100 °C and 35 bar.

According to literature,^[^ ^69^, ^70^ ^]^ a total of 2.11 kg benzene, 0.88 kg propylene, and 0.76 kg ethylene are required to produce 1 kg PO, while 2.54 kg styrene is produced at the same time. Average emissions are 1.4–3.0 g SO_2_/t styrene, 124–162 g NO_*x*_/t styrene, 4–8 g CO/t styrene, 2–3 g dust/t styrene, and 2–3 g NMVOC/t styrene. Relevant wastes are tars and heavy residues from the distillation steps.

#### 2.1.4 Cumene PO Process

The cumene PO process involves the oxidation of cumene (**10**) with air to form cumene hydroperoxide (**11**) at 80 °C and 42 bar (Scheme 1D). The contact with solutions of caustic soda or potassium hydroxide removes acidic byproducts and separates the organic phase from the aqueous one. Propylene is then epoxidized by mixing it with cumene hydroperoxide in a ratio of 1:1, using titanium silicate as a catalyst.^[^ ^72^ ^]^ The raw product is distilled to remove organic impurities and to recycle propylene. The additionally produced cumene alcohol (**12**) is transformed back to cumene in two steps by dehydration over activated alumina followed by hydrogenation of **13** over a palladium or copper catalyst. To produce 1 kg of PO, 0.87 kg of propylene, 0.05 kg of cumene, and 0.04 kg of hydrogen are required. In Europe, there are no plants for the production of PO via the cumene PO route; however, plants are operated in Japan and Saudi Arabia.^[^ ^64^ ^]^

#### 2.1.5 HPPO Process

The hydrogen peroxide‐to‐propylene oxide (HPPO) process is considered as a mild and innovative process (Scheme 1E). It was initially developed on a small scale in Italy at the end of the 1970s and has been used in commercial plants worldwide since 2008.^[^ ^64^, ^73^, ^74^ ^]^ The first plant was commissioned in Ulsan, South Korea, by SKC with Evonik/Uhde Technologies,^[^ ^75^ ^]^ closely followed by the first plant in Europe, built by BASF and Dow in Antwerp.^[^ ^76^ ^]^

In the HPPO process, propylene is oxidized using a titanium silicalite catalyst and hydrogen peroxide.

The epoxidation of propylene is highly exothermic (–213 kJ mol^−1^) and therefore requires efficient cooling. Both Evonik and BASF/Dow use a titanium silicalite (TS‐1) as a catalyst to activate H_2_O_2_. Epoxidation in the BASF/Dow process takes place at less than 90 °C and at pressures of around 30 bar in a tube bundle reactor. Also, a two‐step reactor system is used, which enables an almost quantitative conversion of 99% hydrogen peroxide and PO selectivities of up to 94%.^[^ ^76^ ^]^ On the other hand, the Evonik process takes place at temperatures below 60 °C and at a pressure of around 25 bar in a trickle bed reactor producing small quantities of various marketable propylene glycols as byproducts. Both processes use methanol as solvent, which can be recovered and an excess of propylene, which can also be recovered and recycled. The deactivated catalyst can be regenerated.^[^ ^68^, ^75^, ^77^ ^]^

The HPPO process is used to synthesize PO at multiple sites worldwide. One of these sites is located in the EU (Antwerp), another site was recently opened in Tiszaújváros, Hungary.^[^ ^66^, ^78^ ^]^ The global capacity for the HPPO process was estimated to be around 1,840 kt a^−1^ in 2019.^[^ ^66^, ^68^ ^]^ The integration of an HPPO plant into an existing industrial park requires a constant supply of hydrogen peroxide on a large scale. For the first European HPPO plant with a capacity of 300,000 t in Antwerp (BASF/Dow), a new hydrogen peroxide plant (BASF/Solvay) with a capacity of 230,000 t a^−1^ was also commissioned. The necessary supply of hydrogen for the production of hydrogen peroxide is generally available at various chemical parks. The waste heat from the HPPO plant can be used on site. At the same time, the chlorine demand, which is generally produced on site, will be reduced and thus also the production volume of NaOH linked to the chlorine production. However, since the corresponding consumption of caustic soda or Ca(OH)_2_ for the dehydrochlorination of chlorohydrin is also eliminated, NaOH availability should not be affected by switching to the HPPO process. The amount of waste at the site is significantly reduced compared to the chlorohydrin process.^[^ ^76^, ^79^ ^]^ For the synthesis of PO using the HPPO process, various data for technical parameters can be found in the literature, which are of the same order of magnitude. Per kilogram of PO, 0.60–0.66 kg of hydrogen peroxide and 0.75–0.78 kg of propylene are required as well as 2.5 kg of steam and 0.24 kWh of electricity.^[^ ^64^, ^80^ ^]^

##### 2.1.5.1 Raw Materials and Recycling

The environmental impact of the raw material supply for PO syntheses is largely linked to the corresponding upstream chains. In all processes, propylene for PO synthesis is generally derived from fossil sources and is obtained by steam cracking or by catalytic cracking of distillation residues from crude oil refining (fluid catalytic cracking). The chlorohydrin process requires 1.29–1.35 kg Cl_2_/kg PO and 0.76 kg propylene/kg PO. Added to this are 48 kg water/kg PO and ≈1.38 kg NaOH or Ca(OH)_2_/kg PO (≈10 wt%).^[^ ^64^, ^66^ ^]^

Hydrogen peroxide used for the HPPO process is usually produced using the anthraquinone process.^[^ ^81^ ^]^ The origin and production of hydrogen also play a role in terms of environmental impact. The methanol used in the HPPO process is regenerated and fed back into the process; the same applies to unreacted propylene.^[^ ^64^ ^]^

##### 2.1.5.2 Direct and Indirect Emissions

For the chlorohydrin process, the wastewater TOC content is 100–400 ppm and adsorbable organically bound halogens (AOX) 30–60 ppm. Biological treatment can reduce the AOX content by over 85%. The main components are monopropylene glycol and 1‐chloro‐2,3‐epoxypropane‐glycerol‐monochlorohydrin.^[^ ^64^ ^]^

Direct emissions occur during the HPPO process as a result of the various distillation and purification steps. Among other things, oxygen and inert waste gases are vented. The treatment of the resulting wastewater also generates emissions. Compared to the chlorohydrin process, the amount of AOX and TOC emissions from the HPPO process is presumably lower, as only water is produced stoichiometrically. Various glycols occur as byproducts, which are biodegradable.

Indirect emissions occur in the HPPO process from the upstream chains of the two reactants propylene and hydrogen peroxide as well as during the synthesis of the used TS‐1 catalyst (only small, nonstoichiometric quantities). The latter is produced from tetraethyl orthosilicate and titanium tetraethanolate based from titanium and silicon tetrachlorides, so that hydrochloric acid is released during the synthesis.^[^ ^82^ ^]^ In the chlorohydrin process, the upstream chains of chlorine and NaOH or Ca(OH)_2_ production must be compared with hydrogen peroxide production.

Palladium is used as a catalyst for the hydrogenation of alkyl anthraquinone in hydrogen peroxide production. Polar molecules with a high bioaccumulation factor such as trioctyl phosphate serve as solvents for the hydrogenated phase. Hydrogenation takes place in nonpolar aromatic solvents, which are harmful to health and in some cases carcinogenic. The alkyl anthraquinone derivatives used are also hazardous to the aquatic environment. During the hydrogenation of the alkyl anthraquinone, hydrogenation of the aromatic solvent and unwanted overhydrogenation of alkyl anthraquinone on the aromatic core instead of the quinoid structure can occur. The resulting products must be separated and new alkyl anthraquinone needs to be added.^[^ ^81^, ^83^ ^]^ According to the best available technique (BAT) reference document on the production of large volume organic chemicals (LVOC BREF),^[^ ^70^ ^]^ the main environmental issues result from the emission of volatile organic compounds such as alkylated benzene derivatives, which are formed during the oxidation step. Residues and solid waste are generated during catalyst recovery. An alternative for the environmentally friendly production of hydrogen peroxide in the future could be the direct oxidation of hydrogen (for a review on the direct synthesis of hydrogen peroxide, see Fierro et al.^[^ ^81^ ^]^).

In addition, the production method of hydrogen is of particular importance for the emissions generated in the upstream chain since different CO_2_ emissions are associated with production via steam reforming of methane, water electrolysis, chloralkali electrolysis, or methane pyrolysis, to only name a few.

##### 2.1.5.3 PO Summary

There is potential for reducing the environmental impact of PO production by switching from the chlorohydrin process to chlorine‐free processes. Four processes have already been established for the production of PO which, besides from catalyst synthesis, completely avoids the use of chlorine and chlorine‐containing reagents and in which no chlorinated byproducts or waste materials such as HCl or NaCl are produced.

In particular, the HPPO process offers great potential for savings in energy consumption, in the reduction of waste and wastewater volumes, and in raw material requirements.^[^ ^76^, ^80^ ^]^

It avoids chlorine, as hydrogen peroxide (apart from the catalyst) can be produced without chlorine. The process is already established and has been in commercial operation since 2008.

Coproduct processes (SMPO and MTBE process) are already established, meaning that improvements can be achieved using the best available technology.^[^ ^64^, ^70^ ^]^ These routes can also be environmentally and economically beneficial, especially if the construction of two separate plants is considered instead, for example, for the production of PO and styrene from different raw materials.^[^ ^64^ ^]^

The cumene PO process is not relevant in Europe yet, which is why no statements are made here on environmental relief.

Baer et al.^[^ ^64^ ^]^ estimated that in 2015, 38% of the production volume of PO in Europe would be accounted for by the chlorohydrin process, 36% by the SMPO process, 16% by the MTBE process, and 10% by the HPPO process.

### 2.2 Polyurethane (PU)

The production of polyurethanes (PU) consumes a major part of the chlorine produced in Europe (see Section 1.2). Polyurethanes are formed by polyaddition of various diols with isocyanates, which in turn are derived from the corresponding amines and phosgene. In Germany, the synthesis of the respective isocyanate monomers takes place at large integrated sites. The various isocyanates are supplied to mostly medium‐sized companies who use them together with various diols to produce a wide range of industrial and consumer goods.^[^ ^84^ ^]^

#### 2.2.1 Conventional PU Synthesis

**Scheme 2:**
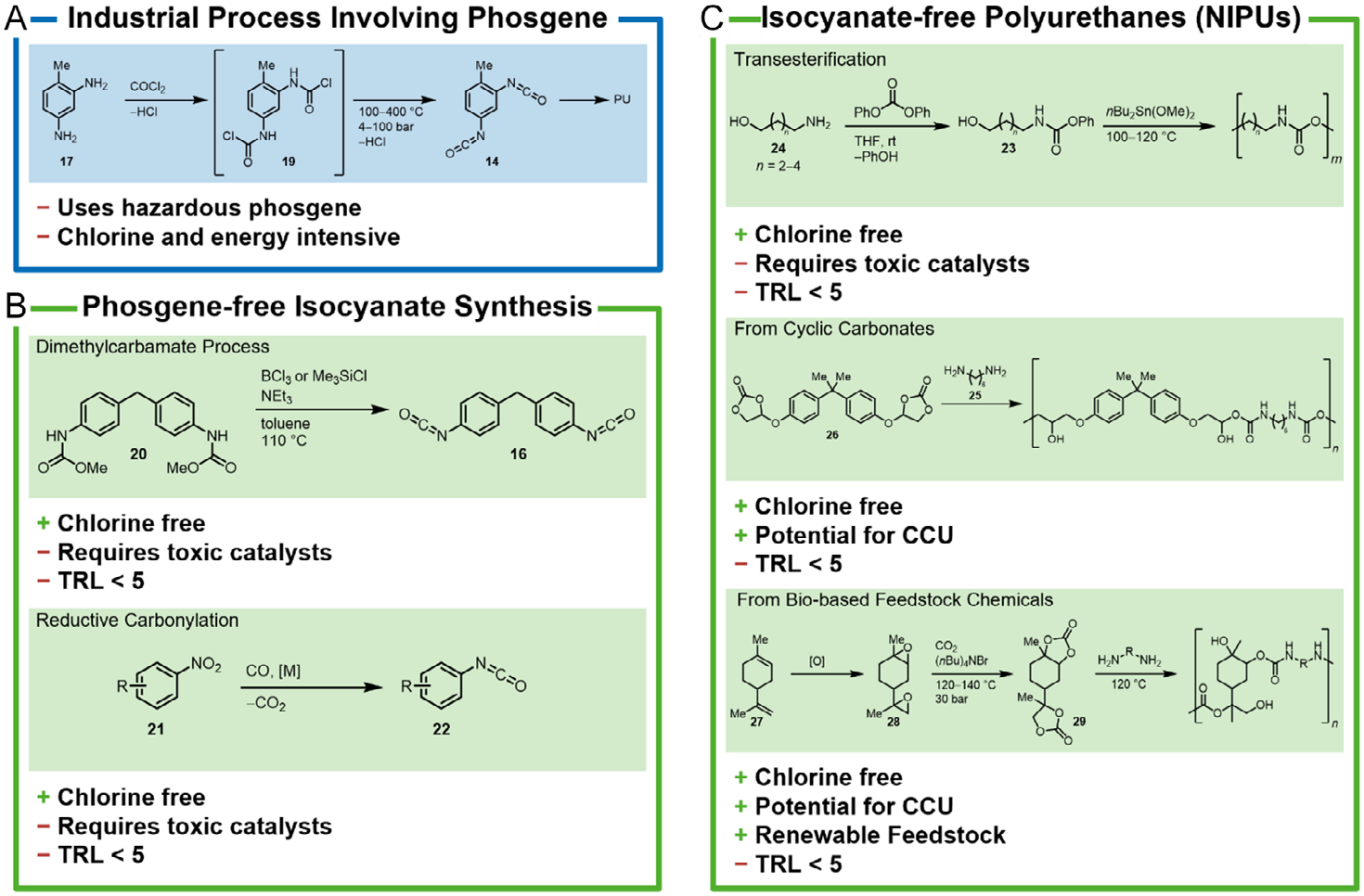
A) Isocyanate synthesis for polyurethanes via phosgene versus B) chlorine‐free isocyanate syntheses and C) isocyanate‐free polyurethanes. TRL = technology readiness level. CCU = carbon capture and utilization.

Isocyanates used for PU production are primarily an isomer mixture of toluene‐2,4‐diisocyanate (TDI, **14**) and toluene‐2,6‐diisocyanate (2,6‐TDI, **15**) as well as methylene diphenyl diisocyanate (MDI, **16**). Isocyanates are produced from the respective amine, for example, toluene‐2,4‐diamine (TDA, **17**) or methylene diphenyl amine (MDA, **18**), by reaction with phosgene (**Scheme** **2**A). In the first step, carbamoyl chloride (**19**) is formed under the release of hydrogen chloride. It then decomposes at elevated temperatures into the corresponding isocyanate releasing a second equivalent of hydrogen chloride.^[^ ^84^ ^]^ The synthesis of isocyanates consumes most of the phosgene produced in Germany.^[^ ^70^, ^85^ ^]^

In general, the phosgenation of TDA (**17**) can be carried out in the gas phase or in the liquid phase, whereas MDA (**18**) is only phosgenated in the liquid phase, which can be carried out as a batch or continuous process. In both cases, an excess (1,5–3 equiv.) of phosgene is mixed with the respective amine. Chlorinated solvents and low reactant concentrations are usually used. In the batch process, the mixing takes place at RT and the mixture is then heated at high pressures until no more hydrogen chloride is released.^[^ ^86^ ^]^ Subsequently, the solvent is removed and the isocyanate is purified by distillation, crystallization, or sublimation. In the continuous process commonly used for aromatic isocyanates, the reactants, solvents, and the amine catalyst are continuously mixed and gradually heated in various reactors to 100–300 °C at 5–100 bar to form the isocyanate,^[^ ^70^, ^86^ ^]^ so that hydrogen chloride is gradually released followed by distillative purification.^[^ ^87^, ^88^ ^]^

The gas phase technology, which was previously used primarily for aliphatic isocyanates,^[^ ^86^ ^]^ has recently also been introduced to aromatic amines.^[^ ^89^, ^90^, ^91^ ^]^ Both the phosgene and the amine used are evaporated and heated separately and then fed into a reactor. Depending on the amine used, the reaction temperature ranges between 280 and 400 °C and a pressure of 4–10 bar. In addition to the reactants, an inert medium containing nitrogen or noble gases and solvents such as dichlorobenzene are present in the reactor. The reaction mixture is then mixed with a solvent to extract the isocyanate, which is then rectified. The remaining components are separated and the solvent is reused.^[^ ^92^ ^]^

For TDI production, this process enables a higher selectivity and thus lower resource consumption, reducing the solvent demand by 80% and the energy demand for distillation by 40%.^[^ ^93^ ^]^ It significantly reduces the excess of phosgene, enabling higher throughputs and thus a downscaling of the plant components. In addition, start‐up and shut‐down times are significantly reduced.^[^ ^70^ ^]^

The hydrogen chloride produced in both processes can be used in various other processes, for example, for the production of ethylene dichloride, a precursor in PVC production. This can lead to very high efficiency in terms of chlorine use.^[^ ^70^, ^86^ ^]^

Detailed environmental impacts of MDI and TDI synthesis are listed in the BAT reference document for the production of LVOC BREF.^[^ ^70^ ^]^ The most important aspects are potential emissions of phosgene, chlorine, and hydrogen chloride. However, these substances should not escape from the closed plant systems during normal operation. In addition, there are emissions of other halogenated compounds as well as polychlorinated dibenzodioxins and dibenzofurans (PCDD/PCDF). These are mainly associated with the production and processing of phosgene. Other emissions into the air include halogenated compounds and VOCs from storage and evaporation. Aqueous wastes are pretreated and mainly contain halogenated compounds such as solvent residues, aromatic nitro compounds (from TDA synthesis), amines from hydrogenation (from TDA synthesis), nitrates, and large amounts of chlorides from phosgenation (MDI and TDI) and condensation (MDA).^[^ ^70^ ^]^

##### 2.2.1.1 Chlorine‐Free Processes

The chlorine‐based synthesis and use of phosgene are associated with a risk potential for humans and the environment, whereas the chlorine production required for phosgene synthesis is energy‐intensive. Therefore, various attempts to establish processes that do not rely on the use of phosgene for the synthesis of polyurethanes were conducted.

In principle, there are two main approaches for a phosgene‐free PU production (Scheme 2B,C): 1) the formation of isocyanates without the use of phosgene or 2) alternative polycondensation reactions that do not rely on isocyanates (so called nonisocyanate polyurethanes, NIPUs).

#### 2.2.2 Phosgene‐Free Isocyanate Synthesis

Several processes for the phosgene‐free synthesis of isocyanates are described in the literature.^[^ ^94^ ^]^ Due to environmental shortcomings and lack of industrial relevance of most of these methods, only the synthesis via methylcarbamates and the reductive carbonylation of nitroarenes will be described here in more detail.^[^ ^3^, ^94^ ^]^

Dimethyl carbonate (DMC) can be used to form carbamates with amines (e.g., methylene diphenyl amine (**20**)), which can be thermally decomposed like their chlorinated congeners to the corresponding isocyanates. However, for the thermal decomposition being selective, the use of boron trichloride or expensive silanes as catalysts is required.^[^ ^95^, ^96^, ^97^, ^98^ ^]^ A more promising approach for a chlorine‐free isocyanate synthesis is the reductive carbonylation of the respective nitro compound (**21**) to form isocyanates (**22**) or carbamates. The reaction takes place at pressures of around 15 bar and at a temperature of 160 °C and is catalyzed by various *d* ^8^–*d* ^10^ transition metal catalysts.

In the absence of alcohols, the isocyanate **22** is formed directly, whereas, when alcohols are present, the corresponding carbamates are formed, which can subsequently be transformed into the isocyanate via thermal decomposition.^[^ ^99^ ^]^

However, the status of development or commercial use of these processes is unclear. In their tutorial review on sustainable routes to polyurethane precursors, Kreye et al. noted that further research is needed at academic and industrial levels to advance the more environmentally friendly and safer production of polyurethanes and their precursors.^[^ ^94^ ^]^ So far (as of 2024), no information on pilot projects has been disclosed.

#### 2.2.3 Isocyanate‐Free Polyurethanes (NIPU)

The second approach to avoid phosgene in the synthesis of polyurethanes is to completely avoid isocyanates as intermediates and to produce so‐called NIPUs (Scheme 2C). Three isocyanate‐free processes are particularly promising, but are not commercially used yet. They have been covered in a review by Blattmann et al.^[^ ^100^ ^]^ and include the following.

1 Polycondensation of linear difunctional carbamates (**23**), that is, derived from diphenyl carbonate (DPC) and aminoalcohols (**24**). Polycondensation takes place at 100–150 °C in the presence of tin catalysts.^[^ ^101^, ^102^ ^]^
2 Polyaddition of diamines (**25**) and cyclic carbonates (i.e., **26**). The latter can be obtained from epoxides and CO_2_. The obtained polymers have chain lengths comparable to those of conventional isocyanate‐based PU polymers. Also, no volatile byproducts such as phenol or ethylene glycol are formed during polymerization. The higher polarity of the resulting hydroxyurethanes results in reduced solubility in most organic solvents but allows for processing from aqueous emulsions.^[^ ^103^ ^]^ In the area of paints and varnishes, isocyanate‐free hydroxyurethanes are commercially available as hybrid polymers. They are characterized by processability on moist surfaces, better adhesion, and better resistance to environmental influences compared to conventional PU varnishes.^[^ ^104^, ^105^ ^]^
3 Bio‐based polyurethanes: A special form of NIPU's are bio‐based polyurethanes. In this case, the starting materials for urethane synthesis are obtained from renewable raw materials, such as fatty acids or terpenes. These can be epoxidized and transformed to cyclic carbonates.^[^ ^106^, ^107^, ^108^, ^109^ ^]^ Mülhaupt and co‐workers developed a NIPU synthesis starting form limonene (**27**) via epoxidation to **28** followed by carboxylation using CO_2_ and quaternary ammonium salts as catalyst. The corresponding cyclic carbonate (**29**) can then be reacted with diamines to form polyurethanes.^[^ ^110^ ^]^

##### 2.2.3.1 Comparison of Environmental Aspects

The substitution of phosgene in the production of isocyanates for polyurethanes offers enormous saving potential in terms of chlorine demand if used on a large scale. However, chlorine‐free PU syntheses are thus far exclusively described at R&D scales. Information on raw material requirements and recycling, waste water and waste, direct and indirect emissions, as well as energy use and efficiency cannot be provided as the relevant parameters are not given in the literature and would also vary greatly for the various precursors and synthesis options described above. A comparison with the conventional and commercially established processes for the production of TDI and MDI is therefore not possible.

Depending on the synthesis route, NIPUs have special chemical properties that make them advantageous for certain applications compared to conventional PU. The synthesis of NIPUs also makes it possible to bind CO_2_ during the synthesis or to rely directly on renewable raw materials and could thus contribute to the defossilization of polyurethane polymers.^[^ ^94^, ^100^, ^106^ ^]^

Furthermore, avoiding isocyanates is expected to result in an increase in occupational safety, as isocyanates are classified as skin and respiratory sensitizers and some isocyanates are also suspected of causing cancer. The handling of isocyanates mainly affects workers, as consumers usually only come into contact with the end product.^[^ ^111^ ^]^

The isocyanate process produces large quantities of HCl. Due to its high purity, the hydrogen chloride can be regarded as a byproduct that is available as a raw material for other processes. Isomeric isocyanates that arise in small quantities during isocyanate synthesis can be used for pharmaceutical applications. Small quantities of chlorinated arenes that arise in the process of isocyanate production remain mainly unused.

##### 2.2.3.2 PU Summary

The chlorine‐free synthesis of polyurethanes is heavily investigated in both academia and industry; however, the new process proposals for the production of PU are not yet technically mature or even competitive compared to the current process. This affects not only economic aspects, but also yields and atom economy.^[^ ^105^ ^]^ The economic barrier for the introduction of a fundamentally new PU production results mainly from the fact that the established processes take place in highly integrated plants and are therefore very cost effective and atom efficient, two properties that the new processes still have to achieve in order to be commercially successful. Furthermore, switching to NIPUs across the board would mean a significant restructuring at the various small sites that currently process isocyanates to suit the respective application. The probability of success is particularly low for the established aromatic di‐ and polyisocyanates (e.g., TDI and MDI). A major factor here is that the expected intermediates and the end product are obtained as bottom products and, due to their composition (mixture of isomers and homologues), are not accessible by distillation or other large‐scale purification operations.

Therefore, no large‐scale industrial developments are expected in the foreseeable future. However, the production of special polyurethane polymers that are produced and used in relatively small quantities is expected to be switching to phosgene‐free processes in the future as those promise lower requirements in terms of plant safety compared to phosgene‐based processes.^[^ ^61^ ^]^

### 2.3 Epichlorohydrin (ECH)

ECH (**30**) is a base chemical that is mainly used for the production of epoxy resins used, for example, for wind blades due to their light weight and high durability.^[^ ^112^ ^]^

#### 2.3.1 Allylchloride Process

**Scheme 3:**
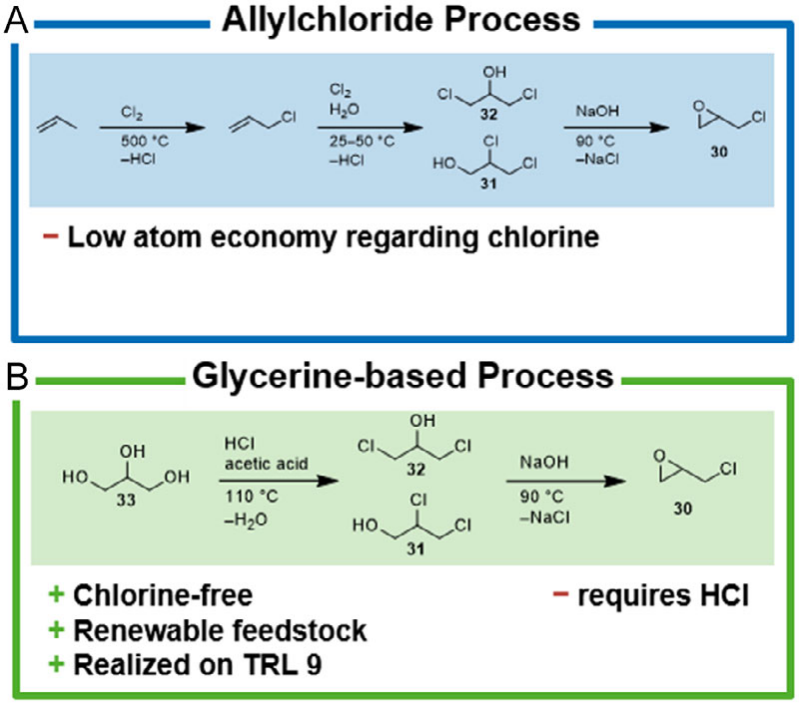
A) Conventional epichlorohydrin synthesis and B) glycerine‐based process. TRL = technology readiness level.

The conventional process for producing ECH follows a three‐stage synthesis starting from propylene (**Scheme** **3**A).^[^ ^113^ ^]^ It involves first the radical chlorination of propylene at 500 °C to produce allyl chloride and hydrogen chloride as a byproduct in a stoichiometric ratio. In addition, other chlorinated propanes and propylenes are formed, which can only be used to a limited extent. After a purification step, the allyl chloride is reacted at 25–50 °C with hypochlorous acid generated in situ to form 1,2‐ and 1,3‐dichloropropanol (DCPol, **31** and **32**) also producing 1,2,3‐trichloropropane as byproduct. The dichlorinated products are purified and mixed with a base (usually aqueous NaOH) at around 90 °C to form the epoxide while also producing NaCl in stoichiometric amounts.^[^ ^113^ ^]^ With 25%, the atomic efficiency of this process is low with respect to the chlorine.^[^ ^113^, ^114^ ^]^

In Germany, ECH based on allyl chloride is produced, for example, by INOVYN in Rheinberg^[^ ^115^ ^]^ and Raschig in Ludwigshafen. The low atom economy of the allyl chloride process and the resulting large amounts of waste (byproducts, waste water, slag) have driven the development of new processes for the production of ECH. The synthesis starting from glycerine receives special economic and ecological attention and will be described here in more detail.

#### 2.3.2 ECH Synthesis from Glycerine

In the first step, two of the hydroxy groups of glycerine (**33**) are chlorinated using hydrogen chloride (Scheme 3B). Carboxylic acids (e.g., acetic acid) serve as catalysts by forming an intermediary acetoxonium species to prevent overchlorination of glycerine resulting in a good selectivity.^[^ ^116^, ^117^ ^]^ The hydrochlorination takes place at 110 °C in a bubble column where hydrogen chloride flows through liquid glycerine. DCPol is then separated from the resulting water, the catalyst, and other diols in a distillation process; the latters can be fed back into the reactor. After purification, the DCPol is reacted with aqueous NaOH at 90 °C, producing ECH (**30**) as well as stoichiometric amounts of aqueous NaCl in concentrations of up to 30%.^[^ ^118^ ^]^

This process is already used in commercial plants, either as a supplement to the existing allyl chloride process or as standalone plants. Glycerine ECH plants can be found in Germany and other countries, for example, in Leuna,^[^ ^119^ ^]^ in Ústí nad Labem, Czech Republic,^[^ ^120^ ^]^ and in Map Ta Phut, Thailand.^[^ ^121^ ^]^ In Tavaux, France, a plant is operated by INOVYN, which produces ECH from both raw materials, propylene and glycerine.^[^ ^122^ ^]^

##### 2.3.2.1 Raw Materials and Recycling

The allyl chloride process requires allyl chloride obtained from propylene, NaOH, and chlorine. Various organic carboxylic acids (e.g., acetic acid) serve as catalysts in the hydrochlorination process, which can be easily separated and are not limited in their availability.^[^ ^123^ ^]^ ≈90 m^3^ of water are needed per ton of ECH. In comparison, the water demand of the glycerine‐based process is reduced to less than 30 m^3^ t^−1^ ECH.^[^ ^120^ ^]^

The glycerine‐based process requires glycerine, hydrogen chloride, catalysts, and NaOH. Glycerine can be obtained from fossil sources as well as plant or renewable raw materials. Renewable raw materials include glycerine from vegetable fats, for example, sunflower and rapeseed oil, but also palm oil, which are currently mainly used for the production of biofuels, where glycerine is formed as a byproduct in the transesterification of fats to fatty acid methyl esters.

In Germany, used cooking oil, rapeseed, and palm oil are the most important raw materials for biodiesel production.^[^ ^124^ ^]^ Glycerine from used cooking oil, that is, already used vegetable oils or animal fats, is also used and classified as waste, and is therefore a positive example of material recovery and recycling. If glycerine is obtained from renewable sources that also bind CO_2_ during the growth phase, ECH can be viewed as a carbon sink.

Used chlorine and NaOH solution in the allyl chloride process are obtained by chloralkali electrolysis. To regenerate both, the formed NaCl could be fed back into the electrolysis after treatment.

In the glycerine‐based process, hydrogen chloride is used instead of chlorine. HCl can be obtained as a byproduct of many chemical processes and thus does not necessarily increase the primary chlorine demand.

##### 2.3.2.2 Direct and Indirect Emissions

The production of ECH using the glycerine process primarily generates indirect emissions during the generation of steam and electricity, depending on the primary energy sources used. Electricity‐related emissions particularly affect the energy‐intensive chloralkali electrolysis. Due to the greater atom efficiency with regard to chlorine (50% instead of 25%), the proportion of electricity‐related emissions is reduced compared to the conventional process. A further reduction of indirect emissions is achieved when HCl is obtained and recycled from other processes.

Compared to the conventional synthesis from propylene, the share of renewable energy sources in total energy consumption (both material and energy) is increased significantly, primarily through the use of bio‐based glycerine and the resulting savings in fossil energy in the upstream production of propylene and chlorine.^[^ ^120^ ^]^ Overall energy requirements are halved and material and water requirements are roughly cut by two thirds. However, the environmental product declaration may not account for all environmental issues associated with upstream plant‐based glycerine production such as land use and loss of biodiversity.^[^ ^120^, ^125^ ^]^

A reduction in greenhouse gas emissions in the upstream chain of glycerine production can be reduced by around 50% if biogenic waste and residues are used. When using rapeseed or palm oil, the greenhouse gas reduction is 30% or less compared to propylene.^[^ ^126^ ^]^

Direct emissions from the glycerine process result from the combustion of byproducts for in‐process energy generation and recovery of HCl (91 kg t^−1^ ECH). Aqueous waste mainly contains NaCl and small amounts of organic contaminants. Other hazardous wastes in the glycerine process are slag residues (47 kg t^−1^ ECH) from distillation, which are burnt and emissions of toxic substances into the air, water, and soil.^[^ ^120^ ^]^

According to the environmental product declaration of Spolchemie's glycerine‐based ECH,^[^ ^120^ ^]^ the glycerine process allows a reduction of the global warming potential (GWP) by 83%, of the ozone depletion potential by 78%, of the acidification potential by 36%, and of the photochemical ozone formation by 62% compared to the allyl chloride process.

##### 2.3.2.3 ECH Summary

Switching to a glycerine‐based production process can lead to environmental relief on several levels.^[^ ^120^, ^125^ ^]^ Direct and indirect emissions, (fossil) raw material requirements, and waste quantities are reduced, while energy efficiency and atom economy increase. The quantity of hazardous byproducts and waste is also reduced significantly. Glycerine‐based ECH still requires chlorine in form of hydrogen chloride, ideally obtained as a byproduct of integrated processes. Compared to the conventional process, the atom economy with regard to chlorine is doubled to 50%. However, in the elimination step, NaCl is still produced as a byproduct, which can be fed back into a coupled chloralkali electrolysis due to the high concentration obtained. The glycerine process is established and highly developed, for example, at INOVYN in Tavaux.^[^ ^122^ ^]^ The prerequisite for ECH from glycerine is the long‐term availability of glycerine from sustainable biogenic sources. However, with the ban on the sale of petrol and diesel vehicles (except e‐fuel‐powered) in Europe from 2035,^[^ ^127^ ^]^ the availability of glycerine from biodiesel production could be diminished in the future.

### 2.4 Chlorinated Methane Derivatives (CMDs)

Chlorinated methanes are a group of four substances that form a homologous series: chloromethane, dichloromethane, trichloromethane (chloroform), and tetrachloromethane. In Germany, around 300–400 kt of chloromethane derivatives are produced per year.^[^ ^128^ ^]^ They are either used as solvents or reagents (mostly for alkylations) and as raw materials for the synthesis of fluorinated hydrocarbons or silicones. The main reason for their use as solvents is the good solubility of many organic molecules due to their high polarity while being aprotic. Another major advantage is that CMDs are hardly flammable, which results in better safety performance on both small and large scales. In addition, chlorinated solvents are relatively inexpensive compared to the nonchlorinated alternatives with similar solvent and safety properties.^[^ ^129^ ^]^

Since chloromethanes contain chlorine, there can be no avoidance, only a minimization of the chlorine demand for their production.

The production process for chloromethane derivatives can be divided into two steps. First step is the hydrochlorination of methanol with hydrochloric acid at 300 and 380 °C using CuCl_2_ on Al_2_O_3_ as catalyst to chloromethane^[^ ^130^ ^]^ followed by a step‐by‐step chlorination to dichloromethane, trichloromethane, and tetrachloromethane using elemental chlorine.

According to Ohligschläger et al.^[^ ^131^ ^]^ the chlorine requirement for the respective chemicals is approximately stoichiometric due to the high selectivity of the initial hydrochlorination and further chlorinations as well as the possibility to reuse HCl formed as byproduct. Hence, there is no relevant potential for saving chlorine in the production of chloromethanes. Chlorine savings can thus only be achieved by the reduction in use of chloromethanes.

The largest area in terms of their end uses is in pharmaceuticals, followed by the infrastructure and construction sector, where silicones are increasingly used.^[^ ^132^ ^]^

In the following, the focus is primarily on alternatives to methylation (or alkylation) reactions with chloromethane as well as nonchlorinated solvents. There are currently no alternatives to chloromethane in the production chain of silicones^[^ ^61^ ^]^ to alkylate elemental silicone producing methylchlorosilanes, which are converted into the respective silicones by hydrolysis and polymerization reactions.^[^ ^133^ ^]^ The possible uses of chlorinated solvents in (pharmaceutical) synthesis and of dichloromethane for chromatographic separation are practically endless. For this reason, only a few exemplary reactions and alternatives are discussed here. Further alternatives are listed in a current review by Jordan et al.^[^ ^129^ ^]^

#### 2.4.1 Chlorine‐Free Alkylations

Chloromethane is often used as a reagent for the methylation reactions, whereas the presence of other leaving groups than chlorine is required to use alternative reagents for methylation. Many of these reagents have a high‐risk potential and may only be used under special reaction conditions. In a review, Chen has compiled the most common reagents for laboratory use (sometimes further reaction steps are necessary, e.g., reduction of the methyl group after carbonylation with formaldehyde).^[^ ^134^ ^]^ For alkylations other than methylation, many synthetic methods are available in addition to the corresponding alkyl chlorides. A comparative analysis of individual processes in certain fields of application is not possible due to the large number of processes.

#### 2.4.2 Alternative Solvents

In order to illustrate the proportion of chlorinated solvents in medical‐pharmaceutical research, Jordan et al. examined the number of reactions using chlorinated solvents in the publications of the Journal of Medicinal Chemistry in 2009 and compared them to the number of reactions using chlorinated solvents in 2019.^[^ ^129^ ^]^ For 2009, a total of 3,282 reactions were found in 129 studies. Chlorinated solvents accounted for 19% of all solvents used; first and foremost was dichloromethane with a total of 18% of all solvents. The same study showed that in 2019 only a minor reduction in chlorinated solvents by 1% was observed, with dichloromethane still in first place (17%). The top 25 solvents used were exclusively organic solvents; water was not among them.

When choosing a solvent for a given reaction in an industrial setting, many criteria must be met. These include, above all, technical criteria such as compatibility with the process and the ability to dissolve the substances used. But health and safety aspects play also an important role. In addition, the solvent used should have a good balance between the social benefits of the product, the resulting environmental impacts and economic costs.^[^ ^135^ ^]^ In their study, Jordan et al. discussed alternatives to chlorinated solvents for a total of 29 different reactions and also separation techniques such as chromatography.^[^ ^129^ ^]^ In order to aid the selection of a suitable solvent, a number of tools have been developed in the past. One example is the ACS Solvent Selection Tool, in which various reaction parameters can be entered, thus limiting the selection of suitable solvents for a reaction.^[^ ^136^ ^]^

#### 2.4.3 CMD Summary

A variety of alternative reagents are available for chlorine‐free alkylation reactions. However, the majority of electrophilic alkylation reagents is just as problematic as the corresponding alkyl chlorides in terms of toxicity. Chloromethanes are widely used as solvents, particularly in pharmaceutical chemistry. When developing new processes, the potential for substituting chlorinated solvents should be exploited. Here artificial intelligence might also be helpful in the future, to aid in choosing solvents with a better ecological profile.^[^ ^61^ ^]^

### 2.5 (Poly)Tetrafluoroethylene ((P)TFE)

Polytetrafluoroethylene (PTFE), also commonly referred to as Teflon, is synthesized from the corresponding monomer tetrafluoroethylene (TFE, **34**). TFE and PTFE are perfluorinated alkyl substances (PFAS) and should thus be avoided as far as possible due to the concerning properties of this compound class.^[^ ^137^ ^]^ Restrictions on PFAS via European chemicals law are currently under discussion.^[^ ^138^ ^]^

Various commercial processes are available for the synthesis of the monomer TFE. These include the reaction of tetrafluoromethane in an electric furnace, the dechlorination of 1,2‐dichlorotetrafluoroethane or debromination of 1,2‐dibromotetrafluoroethane with metals (mostly zinc), and the thermal decomposition of trifluoroacetic acid to TFE, hydrogen fluoride, and CO_2_.^[^ ^139^ ^]^

#### 2.5.1 TFE from Pyrolysis of Chlorodifluoromethane

**Scheme 4:**
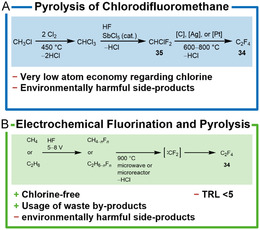
A) Pyrolysis of chlorodifluoromethane and B) chlorine‐free electrochemical fluorination and pyrolysis. TRL = technology readiness level.

The most commonly used process is the synthesis from fluoroform or chlorodifluoromethane (R‐22, **35**) by pyrolysis at 600–800 °C which is described here in more detail (**Scheme** **4**A).^[^ ^140^ ^]^ Chloromethane (see Section 2.4 for details on chloromethane production) is chlorinated two times to form chloroform, followed by a halogen‐fluoride exchange with hydrogen fluoride in the presence of antimony halogenide catalysts to form chlorodifluoromethane (R‐22, **35**), which is then subjected to pyrolysis to yield TFE (**34**).^[^ ^140^ ^]^

The pyrolysis process involves passing gaseous chlorodifluoromethane (**35**) through a reactor containing platinum, silver, or carbon at reduced or atmospheric pressure to pyrolyze TFE at temperatures between 600 and 800 °C. The conversion of chlorodifluoromethane to TFE is low with only 28% at a selectivity of 83% but can be increased to 60–80% using steam or superheated steam (700 °C) while maintaining high selectivities of up to 93%. After pyrolysis, the product gas is washed with water for cooling and removal of HCl followed by a second and third wash with NaOH and concentrated sulfuric acid for drying. The complex product mixture is then separated by distillation in the presence of stabilizers such as limonene or other terpenes to prevent autopolymerization. High‐boiling fractions of the remaining crude reaction mixture are further processed to recover unreacted chlorodifluoromethane and valuable byproducts (e.g., hexafluoropropylene). Other byproducts are HCl, HF, perfluorocarbons, and chlorofluorocarbons.^[^ ^139^, ^140^ ^]^ Overall, this process is very energy intensive since the production of one ton of TFE requires up to five tons of chlorine and over 10,000 kWh of electricity.^[^ ^141^ ^]^

#### 2.5.2 Chlorine‐Free Processes

For a chlorine‐free synthesis of fluorine‐containing molecules, fluorine must be introduced into the molecule by other means than chloride‐fluoride exchange. Thus, only two methods remain available for the synthesis of organic fluorine compounds from nonfluorinated feedstock chemicals.^[^ ^140^ ^]^

1 Substitution of hydrogen in hydrocarbons with fluorine, high‐valent metal or non‐metal fluorides, or by electrochemical fluorination (ECF, also known as Simons process).
2 Addition of fluorine, hydrogen fluoride, or reactive nonmetal fluorides to olefins.

Since the latter method is not applicable for the synthesis of TFE, electrochemical fluorination (ECF) to synthesize the starting materials for TFE synthesis is described here in more detail. For this purpose, hydrocarbons (e.g., methane) are electrolyzed in anhydrous HF without producing free fluorine gas. The hydrogen obtained can be used to provide thermal energy in the further course of the process.

The fluorination reaction takes place by the formation of higher nickel fluorides at the electrode surface and runs further via a radical mechanism on the nickel electrodes that are operated at current densities of 10–20 mA cm^−^^2^.^[^ ^142^ ^]^ The selectivity of the fluorination decreases with increasing number of carbon atoms in the molecule, so small hydrocarbons are preferably used. Perfluorinated^[^ ^143^ ^]^ and partially fluorinated^[^ ^144^ ^]^ hydrocarbons can then be transformed to TFE by pyrolysis. Although evidenced in patent literature,^[^ ^145^ ^]^ to the best of our knowledge, the pyrolysis of per‐ and partially fluorinated alkanes produced from ECF has not been implemented at industrial scale.

##### 2.5.2.1 Raw Materials and Recycling

For the conventional route to TFE starting from methanol, a total of four equivalents of chlorine and two equivalents of hydrogen chloride are needed to produce one equivalent of TFE from methanol, none of which remain in the final product. A total of eight equivalents of hydrogen chloride are released during the synthesis.

Raw materials for ECF followed by pyrolysis are mainly of fossil origin and do not require chlorine or hydrogen chloride. Short‐chain hydrocarbons currently originate mainly from petroleum and are obtained by steam cracking or steam reforming. Hydrogen fluoride is required as a fluorine source, which is obtained from the reaction of fluorspar with sulfuric acid. To what extent byproducts of the alternative processes can be recycled or used in other processes is not yet clear.

##### 2.5.2.2 Direct and Indirect Emissions

A comparative live cycle assessment between microwave‐induced pyrolysis of fluoroform from ECF to TFE and the incineration of flouroform (currently state‐of‐the‐art treatment of waste byproducts of ECF) as well as the classic route to TFE via chlorodifluoromethane (R‐22) has been published by Mierdel et al.^[^ ^144^ ^]^ In terms of environmental performance, the incineration of fluoroform results in the lowest environmental impact; however, also no fluoromonomers can be obtained as it is simply destroyed to HF and CO_2_.

The environmental impact of microreactor pyrolysis of fluoroform from ECF to TFE is however significantly reduced compared to the conventional route via chlorodifluoromethane (R‐22). Greenhouse gas emissions are about half as high at around 60 kg CO_2_‐equiv. per kg fluoroform, as is the use of fossil resources at around 0.65 kg oil‐equiv. Emissions in the human toxicity category are reduced by almost 90%, and the ozone depletion potential is reduced by a total of four orders of magnitude to less than 8 × 10^−7^ kg CFC‐11‐equiv.^[^ ^144^ ^]^

Even in the alternative process, unusable byproducts/waste from ECF and pyrolysis must be destroyed in accordance with the applicable regulations. However, no data is available on the types and quantities of direct emissions into the air and other compartments. The high proportion of byproducts in microreactor synthesis poses the risk of emissions with high greenhouse potential.

The cumulative energy demand for both processes has been calculated to be 79.5 MJ for the route via chlorodifluoromethane (R‐22) and 45.1 MJ for the route via fluoroform prepared by ECF.^[^ ^144^ ^]^

##### 2.5.2.3 TFE Summary

Pyrolysis of fluorinated hydrocarbons from ECF could potentially reduce environmental impacts in the production of TFE and PTFE compared to the conventional route via chlorodifluoromethane (R‐22). However, since these processes are still at a very low level of development, further research is needed before the processes can possibly be established on industrial scale.

Due to the high greenhouse gas impact of the various intermediate and byproducts of ECF, even small amounts of emissions are relevant and must be avoided as far as possible. Therefore, the actual environmental relief cannot be assessed at this point. A more detailed examination and quantification of process emissions at plant level is not yet possible. In addition, there is the general problem that perfluorinated chemicals (PFAS) are persistent in the environment. Therefore, ways should generally be sought to avoid these chemicals altogether and to replace them with other substances.^[^ ^137^ ^]^

### 2.6 Polycarbonate (PC)

Polycarbonates are thermoplastic polyesters with unique properties such as extreme toughness, high transparency, and high heat distortion resistance and can be found in many products of daily life such as electronics and cars.^[^ ^146^ ^]^

#### 2.6.1 Interface Polycondensation of Phosgene

**Scheme 5:**
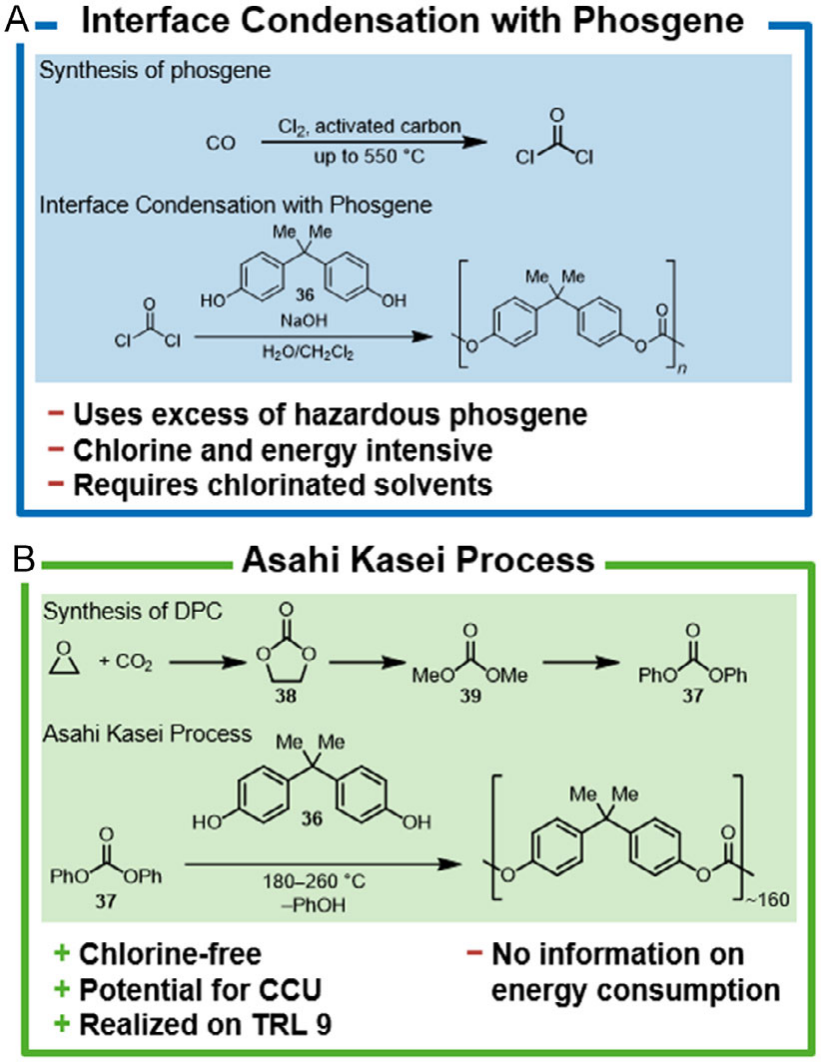
A) Interface polycondensation for PC production versus B) chlorine‐free PC synthesis via transesterification of diphenylcarbonate (Asahi Kasei process). TRL = technology readiness level.

The established process for the production of polycarbonates (PC) is the polycondensation of phosgene with various diols, usually bisphenol‐A (**36**, **Scheme** **5**A).^[^ ^146^ ^]^ The reaction takes place at the interface between an organic and an aqueous phase. Bisphenol‐A is dissolved in the alkaline aqueous phase with NaOH (pH 9–14) as sodium bisphenolate. Phosgene is dissolved in the organic phase usually using dichloromethane or other chlorinated hydrocarbons as solvents. Nucleophilic attack of the phenolate on the carbonyl carbon occurs at the interface and sodium chloride is formed as a byproduct. A 10–20% excess of phosgene in relation to bisphenolate is used to compensate for the hydrolysis of phosgene.^[^ ^146^ ^]^

#### 2.6.2 Transesterification of Diphenyl Carbonates to Polycarbonates in a Melting Process (Asahi Kasei Process)

Due to the high hazard potential of phosgene and the high‐energy input required to produce the necessary elemental chlorine, chlorine‐free processes for the production of polycarbonates have been extensively investigated. One commercially used process is the transesterification of DPC (**37**) with diols (Asahi Kasei process, Scheme 5B).^[^ ^147^, ^148^ ^]^

For this process, **37** is reacted solvent free in a melt with a diol. The same diols as in the conventional process can be used for the polymerization. However, as the melt viscosity of the reaction mixture increases significantly with growing polymer chain length, the removal of phenol by means of evaporation becomes more difficult. Thus, the polymerization is performed in two stages. First, transesterification of DPC with the diol component is performed leading to a prepolymer carbonate with a chain length around *n* = 10, before mechanical stirring becomes impossible. In the original Asahi Kasei process, the obtained highly viscous melt is then fed into a nonagitation polymerization reactor at 260 °C where the prepolymer slowly flows down the channels in the reactor, resulting in good surface renewal allowing for the effective removal of phenol at a pressure of 0.8 mbar. Thus, polycarbonates with up to 50 repeating units can be obtained. Other reactors feature twin‐screw‐type agitation or twin axis bladed reactors or disc‐ring‐type reactors in order to deal with the very high melt viscosity.^[^ ^148^ ^]^ The recovered phenol from the reaction can be used again for DPC synthesis as well as excessive DPC, which is also recovered in the reactive distillation.

DPC can be prepared from CO_2_ and ethylene oxide.

The CO_2_ for DPC synthesis can be sourced, for example, from ethylene oxide synthesis.^[^ ^146^ ^]^ Reaction of CO_2_ with ethylene oxide results in the formation of ethylene carbonate (**38**), which is then transesterified with methanol to form DMC (**39**). This produces stoichiometric amounts of monoethylene glycol, which can be used, for example, for polyester fiber, PET production, or as adsorbent in the Selexol process for CO_2_ capture.^[^ ^149^ ^]^ The same applies to other epoxides which can be used as starting points for DMC synthesis, such as PO, from which propylene glycol is formed as a byproduct.^[^ ^149^ ^]^ DPC (**37**) is then produced via transesterification of **39** to methyl phenyl carbonate, which undergoes self‐transesterification with a second equivalent of methyl phenyl carbonate to form **37** and **39**. In order to shift the equilibrium to the product side and suppress side reactions, the reaction is performed in a multistage reactive distillation column, constantly removing the products from the reaction mixture and shifting the equilibrium to the product side.^[^ ^147^, ^150^ ^]^ The DMC (**39**) produced can be recycled and reintroduced into the process. The same applies to the phenol formed in the polymerization. This results in closed material cycles for MeOH/DMC and phenol/DPC. The CO_2_ used for the carbonate synthesis remains bound in the polycarbonate.

According to Fukuoka et al.^[^ ^147^ ^]^ yields and selectivities of the respective reactions from ethylene oxide and CO_2_ to the precursor molecules ethylene carbonate, DMC, DPC, and monoethylene glycol (MEG) are over 99%. The high yields are achieved by a total of three reactive distillations, since the transesterification reactions have low equilibrium constants. In addition, azeotropic mixtures of methanol and DMC are formed, which are not further separated and lead to high mass flows in the distillation circuit. Reactive distillations usually require a large amount of thermal energy; however, the energy demand is not specified in the literature.

Other routes to aromatic polycarbonates with special material properties have been listed in numerous reviews.^[^ ^151^, ^152^, ^153^ ^]^

##### 2.6.2.1 Industrial Production Plants

In Europe, the first phosgene‐free PC production plant was put into operation in 1998 by GE Plastics (now SABIC) in Cartagena, Spain.^[^ ^148^, ^154^ ^]^ The completely phosgene‐free Asahi Kasei process was reported to account for around 17% (830 kt per year) of global production in 2016. In the meantime, further capacities for the phosgene‐free production of polycarbonates have been created, especially in Asia. Producers are Lihuayi Weiyuan (China), Saudi Kayan (SABIC, Saudi Arabia), Kazanorgsinzes (Russia), Lotte Chemical (South Korea), Lotte Advanced materials (South Korea), and Chimei‐Asahi (Taiwan) with a total capacity of over 1,000 kt per year in 2019. In 2019, ≈20% of all polycarbonate produced globally was based on the Asahi Kasei Process and 80% on other processes, predominantly based on phosgene.^[^ ^146^, ^148^, ^155^ ^]^ At the same time, between 2006 and 2015, four PC production plants that used phosgene as a starting material were shut down.^[^ ^148^ ^]^

In Germany, polycarbonates are produced at two large sites by Covestro in Uerdingen and by Trinseo in Stade.^[^ ^156^ ^]^ Both sites use phosgene for PC production at integrated sites. According to Fukuoka et al. the growing implementation of the Asahi Kasei process to date was mainly due to the fact that the process offers site‐specific economic advantages.^[^ ^148^ ^]^ Compared to phosgene‐based processes, it is stated that the investment costs in plants are only half as high, although up to six reactors are required for the process. However, according to a competing company, the statement about lower investment costs cannot be verified.^[^ ^156^ ^]^

Apart from investment costs, it is further stated that the costs for raw materials are lower because, for example, no BPA is lost in the form of salts (1.65%), high costs for wastewater are avoided, and CO_2_ can be obtained as a raw material at low cost from integration with ethylene oxide production.^[^ ^148^ ^]^

The high process temperatures still require integration at sites with a high excess of thermal energy because it involves several transesterification steps that are thermodynamically unfavorable and energy intensive. Therefore, the production costs based on the Asahi Kasei process are highly dependent on the specific industrial setting and have to be examined in detail.

##### 2.6.2.2 Raw Materials and Recycling

For the production of PC using the conventional process, 0.5 t of phosgene is required per ton of PC. This results in 0.59 t of NaCl as waste and about 10 t of dichloromethane and water are required per ton of PC in the polymerization step.^[^ ^148^ ^]^ Dichloromethane and water are recovered and reused in established processes.^[^ ^157^, ^158^, ^159^, ^160^ ^]^ In addition, 10–100 t of water are used to remove NaCl, unreacted BPA, as well as catalysts (tertiary amines) from the organic phase. It is not known whether this is the actual consumption or whether the wastewater can be partially recovered.

Auxiliary materials are NaOH, tertiary amines as catalysts, hydrochloric acid or phosphoric acid, demineralized water, and depending on the process variant, heptane is used to precipitate PC. It is assumed that the amount of BPA required does not differ significantly from the phosgene‐free process. Differences arise when using phosgene (from CO and chlorine) compared to DPC.

At the Uerdingen site, the saline wastewater from conventional polycarbonate production is fed back into the chloralkali electrolysis after treatment.^[^ ^157^, ^158^ ^]^

An atom efficiency of 100% is specified for the phosgene‐free synthesis of polycarbonates, so that reactants are required in stoichiometric amounts. These are 0.9 kg BPA/kg PC and 0.84 kg DPC/kg PC. Furthermore, 0.74 kg phenol/kg PC are released, which can be fed back into the transesterification to form DPC from DMC.^[^ ^147^ ^]^ However, smaller amounts of the precursor products as well as methanol, CO_2_, and ethylene oxide will probably be lost, increasing the raw material demand accordingly.

Various processes and synthesis routes are available for the production of DMC as a precursor for DPC. These are the oxidative carbonylation of methanol, urea‐based processes, direct synthesis from CO_2_ and methanol, and the Asahi Kasei process based on ethylene carbonate from ethylene oxide (or propylene carbonate from PO) where production and use is associated with great potential hazards. Methanol can in turn be produced from hydrogen and CO_2_, so that coupling to Power‐to‐X technologies could be possible at this point in the process chain.^[^ ^61^, ^161^ ^]^

BPA is obtained by condensing acetone with phenol. Acetone and phenol, in turn, are produced in the Hock process by the reaction of benzene with propylene and oxygen.^[^ ^70^ ^]^

##### 2.6.2.3 Direct and Indirect Emissions

The environmental impacts of conventional PC synthesis are listed in an ecoprofile by PlasticsEurope.^[^ ^162^ ^]^ The GWP (GWP100) for the production of 1 kg PC is 3.4 kg CO_2_‐equiv., the acidification potential is 5.36 g SO_2_‐equiv., the eutrophication potential is 0.72 g PO_4_‐equiv., and particle emissions are 0.32 g. Due to the solubility of dichloromethane in water (20 g L^−1^) and the low boiling point, small amounts are likely to enter the environment via both wastewater and exhaust air.^[^ ^147^ ^]^ According to the BAT conclusions, the emission quantities for dichloromethane are limited to <0.5–1 mg Nm^−^^3^ or 50 g h^−1^, or 15 mg Nm^−^^3^ if techniques are used to recover chemicals (e.g., solvents), provided that the reduction efficiency of the exhaust gas treatment system is ≥95%.^[^ ^163^ ^]^

Direct emissions in the melting process result primarily from the operation of the plant. In contrast to the conventional process using phosgene, there are no emissions from organic solvents and no contaminated wastewater; however, to the best of our knowledge, no quantitative information on direct emissions in phosgene‐free PC processes have been disclosed. Compared to the conventional process, the energy requirement in the Asahi Kasei process is significantly reduced.^[^ ^148^ ^]^ Energy‐related emissions are therefore estimated to be reduced by 2.32 t CO_2_‐equiv. per t PC produced.

In terms of indirect emissions, the upstream chains in particular play a decisive role. This includes, for example, the production of BPA as a reactant in both the phosgene‐based and phosgene‐free processes, which accounts for 61% of the greenhouse gas emissions of conventional PC synthesis.^[^ ^162^ ^]^ In the Asahi Kasei process, the production of the other starting materials DPC and its precursors CO_2_, ethylene oxide, methanol, and their process‐related emissions has to be compared to those in the phosgene‐based process, namely the production of phosgene from chlorine. Suitable life cycle assessment studies for a detailed comparison of the two routes are currently lacking.

##### 2.6.2.4 PC Summary

With regard to emissions, waste, and wastewater quantities, specific information is only available for the phosgene‐based process. It has been claimed that the DPC melting process reduces greenhouse gas emissions and the use of water and solvents.^[^ ^148^ ^]^ As there is no information on the total energy demand of the Asahi Kasei process, no final assessment of the potential for environmental relief can be made here. Detailed life cycle assessments along the entire production chain are hence needed.

The Asahi Kasei process can be performed without chlorine, provided that DMC and DPC are produced without phosgene. Furthermore, the melting process is free of solvents potentially resulting in environmental relief and economic advantages. However, it should be noted that the only peer‐reviewed publication detailing process parameters of the Asahi Kasei process were authored by Asahi Kasei Corporation employees. The process is already widely used commercially, especially in Asia, and offers a promising chlorine‐free alternative to the phase transfer polymerization using phosgene.

## 3 Reactive Ionic Liquids to Improve the Safety of Chlorine Chemistry

Since the early 2000s, ILs have emerged as a versatile tool across various scientific and industrial fields, being of particular interest for the development of greener and safer technologies. Defined as a class of salts with melting points below 100 °C, some ILs even remain liquids at RT. Many ILs exhibit distinctive properties such as a low vapor pressure, high thermal stability, electrical conductivity, and low viscosity. These properties can be specifically tuned by the choice of the cation and the anion, allowing for tailored ILs that meet targeted performance requirements in chemical processes.^[^ ^164^, ^165^, ^166^, ^167^, ^168^ ^]^

In the last decades, it has been shown that the application of ILs can allow, for example, to reduce waste, recycle chemicals, and to avoid harmful reagents.^[^ ^169^, ^170^, ^171^ ^]^ Therefore, ILs have found even industrial applications, such as the biphasic acid scavenging utilizing ILs process, which was introduced by BASF in 2002.^[^ ^172^, ^173^ ^]^ Other technologies utilizing ILs include phosgene‐free chlorination reactions,^[^ ^174^ ^]^ the storage of gases,^[^ ^175^ ^]^ the dehydrochlorination of dichloroethane,^[^ ^176^ ^]^ the adsorption of VCM,^[^ ^177^ ^]^ and the drying of chlorine.^[^ ^178^ ^]^ For a general overview about the applications of ILs see the respective reviews.^[^ ^164^, ^165^, ^166^, ^167^, ^168^ ^]^^]^

Recently, reactive ILs have evolved as a promising compound class for chlorine chemistry. Unlike conventional ILs, which are often designed to be chemically inert, reactive ILs (RIL) such as the trichloride [NEt_3_Me][Cl(Cl_2_)] and the bichloride [NEt_3_Me][Cl(HCl)] are reactive by nature.^[^ ^26^, ^179^ ^]^ Therefore, they are not only able to store chlorine or hydrogen chloride, but can also be used for chlorination or hydrochlorination reactions, respectively. Furthermore, these ILs could provide new flexibility options for the chlorine industry and be utilized as indirect energy storages.

### 3.1 Trichloride Ionic Liquids for Safer Chlorine Storage and Enhanced Reactivity

Although trichlorides are known for over one century, their industrial potential has been explored only recently.^[^ ^26^ ^]^ The trichloride [NEt_3_Me][Cl_3_] is formed by the equilibrium reaction of [NEt_3_Me]Cl with elemental chlorine (Cl_2_, Equation (1)).

(1) $${[{\text{NEt}}_{3}\text{Me}]\text{Cl}+n{\text{Cl}}_{2}⇌[{\text{NEt}}_{3}\text{Me}][\text{Cl}{(\text{Cl}}_{2})}_{n}]$$

By adjusting the amount of chlorine, a range of polychlorides can be produced, from trichlorides ([Cl(Cl_2_)]^−^, *n *= 1, also known as trichlorine monoanions) up to tridecachlorine monoanions ([Cl(Cl_2_)_6_]^−^, *n *= 6), while also mixed systems can be formed with noninteger values of *n*.^[^ ^180^, ^181^ ^]^ It is noteworthy that the bond strength of the chloride and the coordinated Cl_2_ molecule within polychlorides diminishes as the coordination number increases. Consequently, with larger *n* values, the equilibrium is shifted to the left side resulting in an increased chlorine vapor pressure. Most trichlorides, however, maintain a relatively low vapor pressure, making them the most practical polychlorides.^[^ ^180^ ^]^

The formation of the trichloride can be explained by different bonding models like halogen bonding or charge transfer. Usually both concepts interplay within the nature of the chemical bond of these systems. Halogen bonding is basically understood as a noncovalent interaction based mainly on electrostatic interactions between a nucleophilic region of an atom or molecule, such as a chloride ion, and an electrophilic region of a halogen atom, the so‐called σ‐hole, for example, along the Cl—Cl bond. In principle this results in halogen–halogen bonding. However, charge transfer, in which the chloride (Cl^−^) donates electron density into the so‐called σ*‐orbital of the dichlorine molecule (Cl_2_), forming a symmetrical multicentered bonded system like a 3c4e bond, which is predominantly covalent, marks the other side of the bonding situation in such systems.

Anyway, based on this charge transfer situation, the antibonding orbitals of the dichlorine molecule are populated and consequently the Cl—Cl bond in, for example, trichlorides is weaker and longer compared to that of elemental chlorine.^[^ ^180^, ^182^ ^]^ This results also in a slightly enhanced reactivity of trichlorides compared to elemental chlorine.^[^ ^183^, ^184^ ^]^

**Figure 2:**
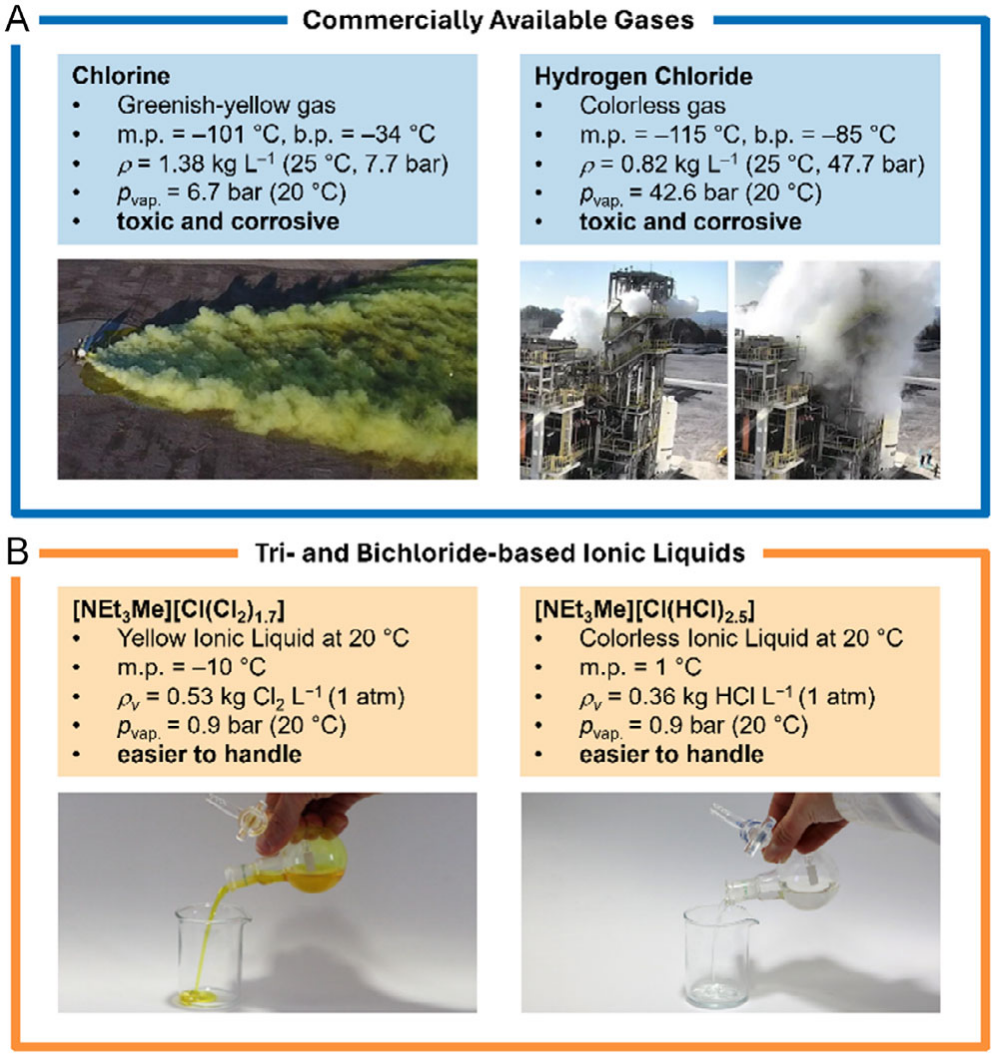
A) Comparison of elemental chlorine and hydrogen chloride with B) the corresponding trichloride and bichloride‐based ILs.^[^ ^4^ ^]^ For liquefied gases, the density (*ρ*) is equal to the storage capacity per liter. Therefore, it is comparable to the storage capacity per liter of the corresponding gases in ILs (*ρ*_*v*_). Pictures used with permission (Copyright: top left: Utah Valley University.^[^ ^213^ ^]^ top right: U.S. Chemical Safety and Hazard Investigation Board.^[^ ^214^ ^]^ Bottom left and right: the authors).

In a systematic study, Riedel and co‐workers investigated the influence of the cation on the physical properties of the corresponding trichlorides including the melting point, the viscosity, and the chlorine storage capacity.^[^ ^185^ ^]^ For example, it was found that [NEt_4_][Cl(Cl_2_)] is a solid at RT, whereas [NEt_3_Me][Cl(Cl_2_)] is a RT IL with a melting point of ≈–10 °C, attributed to the asymmetry of its cation. Among the tested systems, [NEt_3_Me]Cl emerged as the most useful chloride salt, which can store up to 0.79 kg of elemental chlorine per kg chloride salt (**Figure** **2**). Notably, it is readily available from abundant chemicals (NEt_3_ and MeCl) and therefore its costs, when industrially produced on multiton scale, can be estimated to be 4,000–20,000 USD per ton (for details, see the Supporting Information, Section 3). When loaded with chlorine, it forms the corresponding trichloride [NEt_3_Me][Cl(Cl_2_)] which is a reactive IL at RT, making it easy to pump and process within industrial plants. It was also found to be stable for at least two years at RT.^[^ ^185^ ^]^ In contrast to the established pressure liquefaction of chlorine building up a vapor pressure of 6.7 bar at 20 °C, [NEt_3_Me][Cl(Cl_2_)] has a significantly lower vapor pressure of less than 1 bar. Thus, unlike conventional chlorine storage, where even minor mechanical failures can result in significant environmental and safety hazards, trichloride‐based ILs store chlorine securely while greatly reducing these risks.^[^ ^46^, ^185^ ^]^

By applying moderate heat (80 °C) or reduced pressure to the trichloride, the stored chlorine can be released while regenerating the corresponding chloride salt [NEt_3_Me]Cl. Interestingly, also the addition of water to the trichloride results in a release of the stored chlorine while forming an aqueous solution of [NEt_3_Me]Cl, serving as a complementary and simple release method.^[^ ^185^ ^]^

Alternatively, the trichloride [NEt_3_Me][Cl(Cl_2_)] is directly used as chlorination agent sparing the prior chlorine release from the IL. When used as a chlorination agent, [NEt_3_Me][Cl(Cl_2_)] offers the advantage of being much safer and easier to handle than chlorine gas. In addition, this IL can conveniently be dosed which allows better reaction control when an exact reaction stoichiometry is crucial.^[^ ^26^, ^186^ ^]^

In general, the trichloride [NEt_3_Me][Cl(Cl_2_)] shows a reactivity which is similar to that of elemental chlorine.^[^ ^26^ ^]^ For instance, alkenes react in an antiaddition to the corresponding 1,2‐dichloroalkanes while ketones undergo *α*‐chlorination.^[^ ^187^ ^]^ However, in some cases, [NEt_3_Me][Cl(Cl_2_)] can enable reaction pathways that are not achievable under similar conditions with elemental chlorine, given the ionic environment in the IL and the weakened Cl—Cl bond present in the trichloride.^[^ ^183^, ^184^ ^]^

**Scheme 6:**
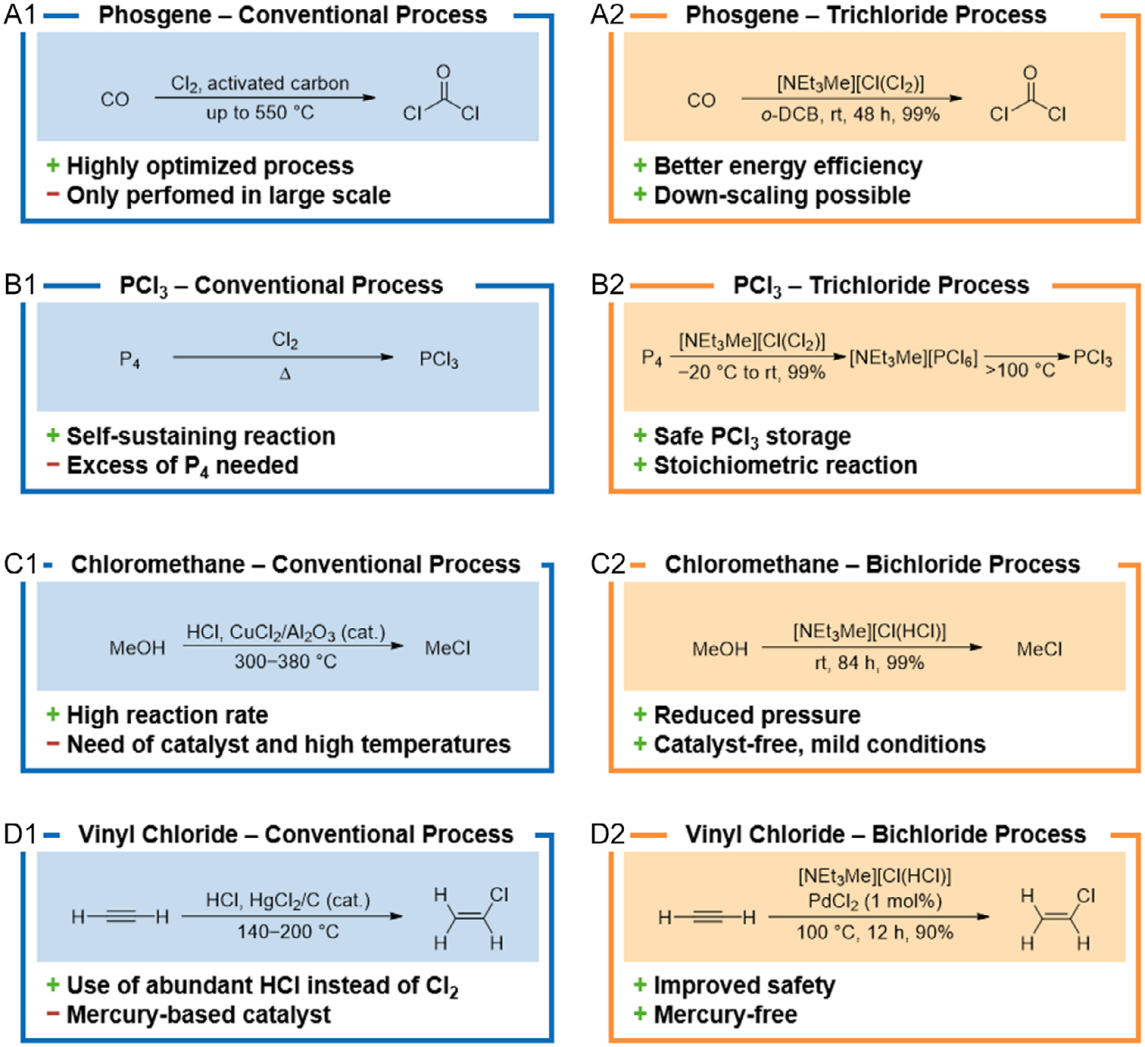
Comparison of established processes involving chlorine or hydrogen chloride (A1–D1) and the corresponding alternatives utilizing bi‐ or trichloride‐based ILs, respectively (A2–D2). *o*‐DCB = *ortho*‐dichlorobenzene.

In this context, the reaction of [NEt_3_Me][Cl(Cl_2_)] with carbon monoxide (CO) to the industrial base chemical phosgene (COCl_2_) has to be mentioned. Traditionally, phosgene is synthesized in large industrial reactors through the gas‐phase reaction of carbon monoxide (CO) and chlorine (Cl_2_) at elevated temperatures (reaching up to 550 °C at reaction hot spots) over activated carbon catalysts (see also above and **Scheme** **6**A1).^[^ ^85^ ^]^ In contrast, utilizing [NEt_3_Me][Cl(Cl_2_)] as chlorination agent in *ortho*‐dichlorobenzene (*o*‐DCB), the synthesis of phosgene can be realized at RT and safely as recently shown by a small lab‐scale demonstration (Scheme 6A2).^[^ ^183^ ^]^

Quantum‐chemical calculations suggest that the reaction pathway involves a chloride‐catalyzed CO insertion into the activated Cl—Cl bond with a relatively low‐energy barrier of 56.9–77.6 kJ mol^−1^, contrasting sharply with the higher activation energy of the uncatalyzed reaction. The mild conditions could reduce the risk of reactor hotspots and minimize byproducts common in the established high‐temperature process. Notably, in this reaction, [NEt_3_Me]Cl serves both as a chlorine storage medium and as a catalyst, potentially enabling continuous phosgene production in a more controlled manner. Overall, this work could contribute to a safer, more energy‐efficient phosgene production, aligning with green chemistry goals by reducing harsh reaction conditions.^[^ ^183^ ^]^

Another chlorine‐derived base chemical that is produced under harsh and inherently dangerous conditions employing elemental chlorine is phosphorus trichloride (PCl_3_), the starting material for countless pharmaceuticals and agrochemicals (Scheme 6B1). Recently, the groups of Riedel and Müller disclosed a novel synthetic approach to phosphorus trichloride using the trichloride‐based IL [NEt_3_Me][Cl(Cl_2_)], which serves as chlorine source and reaction medium.^[^ ^188^ ^]^ Thus, the utilization of chlorine gas could be avoided, improving the safety of the PCl_3_ production. By reaction of white phosphorus (P_4_) with [NEt_3_Me][Cl(Cl_2_)] under mild conditions hexachlorophosphate [NEt_3_Me][PCl_6_] is formed which precipitates as a colorless powder. When heated to temperatures above 100 °C, the hexachlorophosphate releases PCl_3_ gas (Scheme 6B2). Therefore, this compound could be used as a stable storage of PCl_3_, potentially being a safer alternative to PCl_3_ when transported and handled. Notably, [NEt_3_Me][PCl_6_] can be employed as a reagent, showing a reactivity similar to PCl_5_, efficiently transforming carboxylic acids to acyl chlorides and ammonium chloride (NH_4_Cl) to hexachlorophosphazene, which is an intermediate in polymer synthesis. In these reactions, consuming formally PCl_5_ and releasing HCl, a colorless byproduct is formed, which was identified to be the bichloride‐based IL [NEt_3_Me][Cl(HCl)].^[^ ^188^ ^]^

### 3.2 Bichloride‐Based ILs for Hydrogen Chloride Storage and Hydrochlorination Reactions

Hydrogen chloride (HCl) is a base chemical in many industrial processes but poses substantial challenges due to its corrosive, volatile, and toxic nature, especially when large amounts of HCl are stored or transported.^[^ ^57^ ^]^ To deal with this difficulty, Riedel and co‐workers introduced triethylmethylammonium bichloride [NEt_3_Me][Cl(HCl)] as a safer storage solution for pure, anhydrous HCl.

(2) $$\left[{\text{NEt}}_{3}\text{Me}]\text{Cl}+n\text{HCl}⇌[{\text{NEt}}_{3}\text{Me}][\text{Cl}{\left(\text{HCl}\right)}_{n}\right]$$

In analogy to the trichloride system, this bichloride‐based IL is based on the ammonium chloride salt [NEt_3_Me]Cl which reacts with gaseous HCl to the corresponding bichloride [NEt_3_Me][Cl(HCl)], also known as hydrogen chloride chlorate(–i) anion ([Cl(HCl)]^−^, Equation (2)). In contrast to the trichloride anion [Cl(Cl)_2_]^−^, which is formed by halogen bonding (see Section 3.1), the bichloride anion [Cl(HCl)]^−^ exists due to strong hydrogen bond interactions.^[^ ^179^, ^189^ ^]^

An investigation of bichlorides obtained from different ammonium chlorides revealed that the bichloride [NEt_3_Me][Cl(HCl)] shows overall the best physicochemical properties for industrial applications like a low viscosity and a high storage capacity.

The use of [NEt_3_Me][Cl(HCl)] effectively eliminates the need to pressure liquify HCl for storage and simplifies its handling by reducing operational hazards.^[^ ^179^, ^189^ ^]^ Since bichloride‐based ILs exist in an equilibrium with gaseous HCl, they show a low HCl vapor pressure allowing their handling also at air (Figure 2). Therefore, the release of HCl can be realized by either applying heat or vacuum to the system of which the heat‐induced release might be favored for industrial applications. Alternatively, the HCl release is spared using the bichloride‐based IL directly for, for example, hydrochlorination reactions, as the IL provides a liquid reaction medium concentrating high quantities of HCl on a small volume.^[^ ^179^ ^]^ In this way, it is possible to use the bichloride as an easy‐to‐handle and efficient hydrochlorination reagent with a superior safety profile compared to gaseous HCl (Figure 2).^[^ ^5^, ^55^, ^179^, ^190^ ^]^

One important industrial application of gaseous HCl is the synthesis of chloromethane from methanol. The reaction is usually performed at temperatures between 300 and 380 °C using CuCl_2_ on Al_2_O_3_ as catalysts (Scheme 6C1, see also Section 2.5).^[^ ^130^ ^]^ In contrast, the conversion of methanol to chloromethane can be achieved at ambient conditions without the need of a catalyst when [NEt_3_Me][Cl(HCl)] is used as a reagent (Scheme 6C2).^[^ ^179^ ^]^

Another industrially relevant application of [NEt_3_Me][Cl(HCl)] is the production of monomeric VCM, the precursor to PVC (see Section 1.2). VCM is mainly produced from ethylene via 1,2‐dichloroethane (obtained by chlorination or oxychlorination of ethylene) or by the hydrochlorination of acetylene with gaseous HCl.^[^ ^63^, ^191^ ^]^ As acetylene is industrially produced from coal, the acetylene route is important in countries with large coal deposits such as China, the world's largest PVC producer (in the EU, this process is not used anymore).^[^ ^1^, ^63^, ^191^, ^192^ ^]^ The hydrochlorination of acetylene is typically performed by the reaction of acetylene with HCl at 140–200 °C in the presence of HgCl_2_ supported on carbon as catalyst (Scheme 6D1).^[^ ^179^ ^]^

However, as the Minamata Convention calls for the phase out of the industrial use of mercury, there is an urgent need to establish a mercury‐free process for the production of VCM from acetylene.^[^ ^1^, ^193^, ^194^, ^195^ ^]^ Seminal contributions of the groups of Hutchings and Pérez‐Ramírez to the heterogeneously catalyzed hydrochlorination of acetylene revealed that catalysts based on gold or platinum, respectively, are promising alternatives.^[^ ^196^, ^197^, ^198^, ^199^ ^]^ Besides, it was observed that ILs as reaction media can increase the reaction rate of acetylene hydrochlorination and enhance the stability of the employed metal catalyst.^[^ ^194^, ^200^, ^201^, ^202^ ^]^ In this context, Riedel and co‐workers used the IL [NEt_3_Me][Cl(HCl)] serving not only as a reaction medium but also as a hydrochlorination reagent in the presence of PdCl_2_ as catalyst providing VCM in 90% yield under milder conditions (Scheme 6D2).^[^ ^179^ ^]^ In this way, the safety and efficiency of the hydrochlorination of acetylene could be improved while also aligning with efforts to eliminate hazardous mercury catalysts of the PVC production.

HCl is nowadays mainly produced as a byproduct of the chlorine industry, for example, in the synthesis of PU and PC precursors, and is already exceeding its demand.^[^ ^203^ ^]^ The released HCl is either neutralized or regenerated to Cl_2_ by electrolysis or the Deacon process in which HCl is oxidized by molecular oxygen to Cl_2_ using primarily copper catalysts.^[^ ^130^, ^204^ ^]^ Although the electrolysis of hydrochloric acid is a well‐known process, it suffers from corrosion of the equipment and the parasitic evolution of oxygen due to the lower decomposition voltage of water.^[^ ^203^ ^]^

It is also possible to electrolyze gaseous HCl and even though this process is almost as efficient as the Deacon process, it requires the use of special cell set ups and membranes often made from perfluoroalkyl substances (PFAS).^[^ ^205^ ^]^

**Scheme 7:**
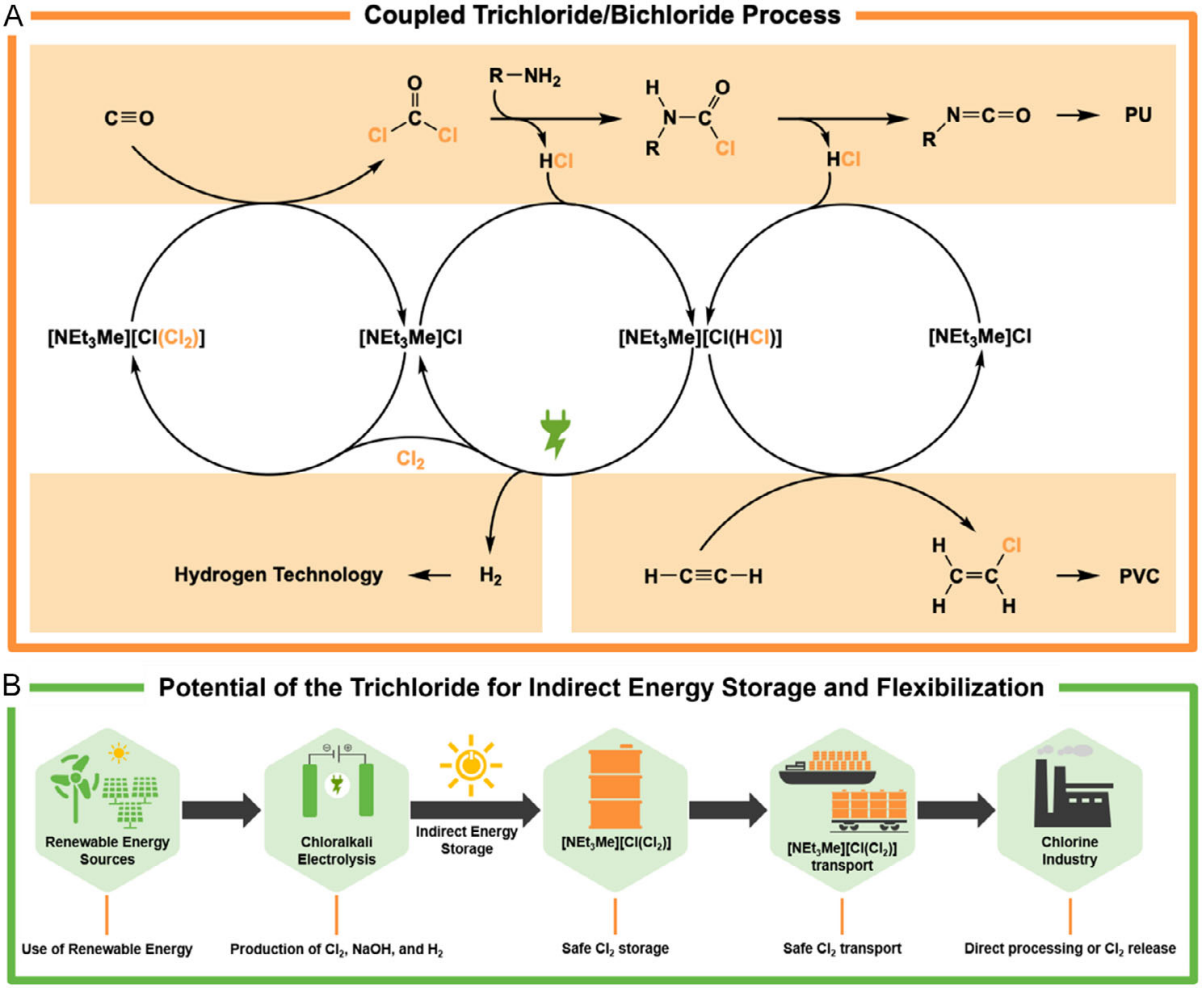
A) Envisioned process combining tri‐ and bichloride‐based ILs and B) potential of the trichloride‐based IL.

Based on these results, a coupled bichloride/trichloride process could be envisioned (**Scheme** **7**A). As described above, [NEt_3_Me][Cl(Cl_2_)] can be reacted with carbon monoxide providing phosgene (COCl_2_) under ambient conditions.^[^ ^183^ ^]^ During this reaction, the unloaded chlorine storage, [NEt_3_Me]Cl, is formed, which is the precursor for both the trichloride [NEt_3_Me][Cl(Cl_2_)] and the bichloride [NEt_3_Me][Cl(HCl)] effectively acting as a bridge between the two systems. The obtained phosgene can be utilized for the synthesis of isocyanates, which are precursors for, for example, PU. In this synthesis, HCl is released as a byproduct which could be captured by [NEt_3_Me]Cl forming [NEt_3_Me][Cl(HCl)]. The bichloride could subsequently be used for the hydrochlorination of acetylene. Alternatively, it is electrolyzed to regenerate the trichloride [NEt_3_Me][Cl(Cl_2_)] while also producing dry hydrogen.^[^ ^179^ ^]^ Therefore, the ILs [NEt_3_Me][Cl(HCl)] and [NEt_3_Me][Cl(Cl_2_)], both derived from the common precursor [NEt_3_Me]Cl, can function as a versatile platform for the safe storage and processing of the essential yet hazardous chemicals HCl and Cl_2_. Notably, this envisioned process would still require fossil‐derived acetylene and the economic and ecological profile of this process has not been elucidated in detail, thus far.

### 3.3 Potential of the Trichloride‐Based Ionic Liquid for the Flexibilization of Chemical Processes and Indirect Energy Storage

The production of chlorine ranks among the most energy intensive chemical processes, in fact, the enormous energy demand is responsible for 67–77% of the total costs of chlorine in Europe.^[^ ^29^ ^]^ Therefore, operating chloralkali electrolysis with renewable energies could significantly reduce the carbon dioxide footprint of the chlorine production. However, renewable energy sources are largely weather dependent, providing an inconsistent power supply. This is in contrast to the chlorine industry which operates processes in a continuous fashion and therefore relies primarily on continuous baseload electricity provided by fossil energy sources. To bridge this discrepancy and enable continuous chlorine production using discontinuous renewable energy, a scalable and durable chlorine storage solution could become a key technology (Scheme 7B).

Currently, “chlorine cannot be easily stored in large amounts” because “the safety requirements for its storage are particularly high.”^[^ ^206^ ^]^ As a result, chlorine is typically only stored for several hours up to two days, restricting the flexibility in chlor‐alkali electrolysis.^[^ ^207^, ^208^, ^209^, ^210^ ^]^ Therefore, realizing an effective chlorine storage solution is inherently linked to addressing the hazards posed by elemental chlorine. In this context, the trichloride‐based IL [NEt_3_Me][Cl(Cl_2_)] offering a safe alternative to the pressure liquefaction of chlorine could become the missing link.^[^ ^185^ ^]^

As the trichloride‐based IL is able to store up to 0.79 tons of chlorine per ton of storage material [NEt_3_Me]Cl, large quantities of chlorine can safely be stored on a relatively small space comparable to that of the established pressure liquefaction.^[^ ^185^ ^]^

Given that the production of one ton of chlorine requires ≈2.6 MWh of electricity (average in Europe in 2023),^[^ ^28^ ^]^ and that 1 t of the loaded chlorine storage [NEt_3_Me][Cl(Cl_2_)] has a chlorine content of 0.44 t per ton of storage material, 1 t of loaded chlorine storage material can indirectly store about 1.1 MWh of energy. In the case of an industrial chlorine plant producing 1000 t of chlorine per day, the energy stored from 2 days of chlorine production would amount to ≈5.2 GWh. To store the chlorine produced within two days using [NEt_3_Me][Cl(Cl_2_)], a storage volume of 3,760 m^3^ would be required that is equivalent to one and a half Olympic swimming pools (one pool contains 2500 m^3^ of water).^[^ ^211^ ^]^ For comparison, the pumped‐storage plant Wehr, with its upper Hornberg basin in Germany's Black Forest, has a storage capacity of 6.0 GWh and a reservoir volume of 4.4 million m^3^, equivalent to about 1,760 Olympic swimming pools, illustrating the high‐energy density achievable with the trichloride‐based IL (for details, see the Supporting Information, Section 4).^[^ ^212^ ^]^

Furthermore, the utilization of the trichloride‐based IL greatly improves the safety of transporting chlorine, especially in countries where the production of chlorine and chlorine‐consuming processes are often not located at the same site. This method reduces the need to transport chlorine in pressurized liquid form via road, rail, or sea, thereby lowering the risk of accidents like the recent one in Jordan.^[^ ^49^ ^]^ After transport, the IL could either be utilized directly for further reactions or heated to release the stored chlorine gas. The unloaded storage material could then be reused in a closed cycle.

Overall, the trichloride‐based ILs could become a pivotal technology in making the chlorine production both more sustainable and safer, aligning the chlorine industry with future energy goals. Furthermore, it can also be used for new business models, as the ability to make the electrolysis process more flexible via the chlorine storage system opens up the possibility of responding more flexibly to the electricity market.

## 4 Summary and Outlook

Chlorine is integral to modern industry and society, forming the foundation for numerous pharmaceuticals, plastics, agrochemicals, and disinfectants. Its importance is further underscored by its role in producing hydrogen chloride, another base chemical that arises as a byproduct in many industrial processes involving chlorine. However, the inherent risks associated with chlorine and hydrogen chloride, such as toxicity and the potential for hazardous accidents, cannot be ignored. The recent accident in Jordan, where a chlorine vessel rupture caused casualties and significant injuries, highlights the ongoing safety challenges. These risks, coupled with the environmental burden of chlorinated byproducts, necessitate a re‐evaluation of chlorine's use and handling in the chemical industry.

Progress toward replacing or reducing chlorine in industrial processes has already yielded notable advancements. For instance, the HPPO process outcompetes the traditional chlorohydrin process, providing a chlorine‐free route to produce PO, a key intermediate in the production of polyols, polyurethanes, and other materials. Similarly, phosgene‐free methods, such as melt transesterification, are increasingly employed in the production of polycarbonates, reducing the reliance on chlorine derivatives. While these innovations offer safer and more environmentally friendly alternatives, upscaling such processes to replace established processes remains a challenge requiring sustained research and development.

In applications where chlorine and hydrogen chloride remain indispensable, ILs present a promising approach to enhance safety. The trichloride‐based ILs [NEt_3_Me][Cl(Cl_2_)] enables the safe storage and transport of chlorine by avoiding the need for pressure liquefaction, reducing the risks associated with handling elemental chlorine. Similarly, the bichloride‐based ILs [NEt_3_Me][Cl(HCl)] allows for the safe storage and transport of hydrogen chloride. Both ILs serve as reagents in chlorination or hydrochlorination reactions, respectively, improving process safety and flexibility. Furthermore, the trichloride‐based IL could be employed as indirect energy storage to streamline chlorine production by chloralkali electrolysis with renewable energies, contributing to a more sustainable industrial framework.

The question of whether chlorine is an essential chemical or a replaceable risk reflects the broader challenge of balancing industrial necessity with sustainability and safety. While chlorine is unlikely to be fully replaced in the next decades, significant advancements in chlorine‐free processes and safer technologies using ILs could redefine the role of chlorine. This could contribute to a transformation of the chemical industry, aligning its practices with evolving environmental and safety standards.

## Conflict of Interest

The authors declare no conflict of interest.

## Author Contributions

**Johannes Schwan**, **Merlin Kleoff**, and **Sebastian Riedel** conceptualized the manuscript. Chapter 1 was written by **Merlin Kleoff**, **Gesa H. Dreyhsig**, and **Patrick Voßnacker**, and revised by all other authors. Chapter 2 is based on a recent report of **Marian Rosental** and **Traute Fiedler**, was written by **Johannes Schwan** and revised by all other authors. Chapter 3 was written by **Merlin Kleoff** and **Gesa H. Dreyhsig** and revised by all other authors. Chapter 4, abstract, and TOC text were written by **Merlin Kleoff** and **Johannes Schwan** and revised by all other authors. The Supporting Information were written by **Patrick Voßnacker** and **Merlin Kleoff** and revised by all other authors.

## Supporting information

Supplementary Material
