# Supplementary material for: Rethinking Chlorine: Essential Chemical or Replaceable Risk?
Source: ChemSusChem. 2025 May 6;18(13):e202402697. doi: 10.1002/cssc.202402697 (PMC12232123; doi:10.1002/cssc.202402697)
Supplement: Supplementary file 1 — Supplementary Material [file CSSC-18-e202402697-s001.pdf]

Supporting Information

Rethinking Chlorine: Essential Chemical or Replaceable Risk?

Johannes Schwan, Merlin Kleoff, Gesa H. Dreyhsig, Patrick Voßnacker, Traute Fiedler, Marian Rosental, Sebastian Riedel*

Calculations

1. Energy Consumption of Chlorine Production

Table S1. Chlorine production capacities, the corresponding power consumption for chlorine production at full usage rate, total energy production, share of power consumption for chlorine production with full usage rate on the in total produced energy, share corrected by actual utilization rate for all chlorine producing countries in Europe.

Country	Chlorine Production Capacities [kt][a]	Power Consumtion [TWh][b]	Energy Production [TWh][c]	Share[d]	Share Corrected by Utilization rate[e]
Austria	77	0.20	69	0.30%	0.20%
Belgium	1074	2.85	96	2.96%	2.03%
Czech Republic	78	0.21	85	0.24%	0.17%
Finland	75	0.20	72	0.28%	0.19%
France	1377	3.65	475	0.77%	0.53%
Germany	5454	14.45	580	2.49%	1.71%
Greece	10	0.03	53	0.05%	0.03%
Hungary	480	1.27	36	3.53%	2.42%
Ireland	11	0.03	34	0.09%	0.06%
Italy	356	0.94	284	0.33%	0.23%
Netherlands	847	2.24	122	1.84%	1.26%
Norway	366	0.97	158	0.61%	0.42%
Poland	404	1.07	180	0.59%	0.41%
Portugal	142	0.38	49	0.77%	0.53%
Romania	208	0.55	56	0.98%	0.67%
Slovak Republic	70	0.19	27	0.69%	0.47%
Slovenia	16	0.04	14	0.30%	0.21%
Spain	412	1.09	292	0.37%	0.26%
Sweden	123	0.33	173	0.19%	0.13%
Switzerland	47	0.12	57.9	0.22%	0.15%
UK	481	1.27	323	0.39%	0.27%
**Total**	**12108**	**32.09**	**3235.9**	**0.99%**	**0.68%**

[a] Chlorine Production capacities at 1st January 2022[1] [b] Calculated for 100% usage rate of the chlorine production capacity and an energy consumption of 2650 kWh/t Cl_2 (weighted average electricity consumption given by Euro Chlor for 2022)[2] [c] Gross electricity production in 2022[3-5] [d] Share of the power consumption of chlorine production at full usage rate on the total produce energy [e] Share corrected by the actual utilization rate of the chlorine production capacities in 2022 (68.5%).[2]

2. Prices of Important Base Chemicals

Table S2. Average price (between September 2018 and September 2019) of base chemicals in Europe in comparison to chlorine.[7]

Chemical	Price [€/t]
Sulfuric Acid	59.32
Nitric Acid	141.14
Chlorine	186.64
Ammonia	256.00
Sodium Hydroxide	265.30
Methanol	364.46
Phosphoric Acid	603.63
Benzene	645.24
Ethylene	1001.76
Acetylene	1545.40
Bromine[a]	3372.45
Iodine	23761.22

[a] Price is given for southeast Asia as no data for Europe is available.

3. Estimated Costs of the Chlorine Storage $[NEt_3Me]Cl$

The chlorine storage material $[NEt_3Me]Cl$ can be produced from the abundant materials triethylamine (NEt_3, 2755 USD/t (2023)) and chloromethane (MeCl, 409 USD/t (2023)).[8–10]

1 t of $[NEt_3Me]Cl$ (MW = 151.68 g/mol) corresponds to an amount of substance of

$$n_{[\mathrm{NEt3Me]Cl}} = \frac{m_{[\mathrm{NEt3Me]Cl}}}{\mathrm{M}_{[\mathrm{NEt3Me]Cl}}} = \frac{1{,}000{,}000\ \mathrm{g}}{151.68\ \mathrm{g/mol}} = 6592.83\ \mathrm{mol}$$

Assuming a yield of 100%, for its synthesis 6592.83 mol of NEt_3 (MW = 101.19 g/mol) having a mass of 0.66713 t and 6592.83 mol of MeCl (MW = 50.49 g/mol) having a mass of 0.33287 t are required. The costs of the required mass of materials are

$$C_{\mathrm{NEt3}} = 0.66713\ \mathrm{t}\ \times 2755\frac{\mathrm{USD}}{\mathrm{t}} = 1837.94\ \mathrm{USD}$$

and

$$C_{\mathrm{MeCl}} = 0.33287\ \mathrm{t}\ \times 409\frac{\mathrm{USD}}{\mathrm{t}} = 136.14\ \mathrm{USD}$$

Therefore, the costs of the starting materials for the production of 1 t of $[NEt_3Me]Cl$ are

$$C_{\mathrm{tot}} = C_{\mathrm{NEt3}} + C_{\mathrm{MeCl}} = 1974.08\ \mathrm{USD}$$

The real production costs will be influenced by the required solvents, personnel costs, cleaning, waste management, and energy, which are highly dependent on the production scale and consequently hard to estimate. As a rule of thumb, “in chemical plants, raw-material costs are usually in the range of 10 to 50 percent of the total product cost.”[11] Therefore, we assume that the real costs of $[NEt_3Me]Cl$ will range between 4,000 and 20,000 USD/t.

4. Trichloride as Indirect Energy Storage

For the production of 1 t of Cl_2 by chloralkali electrolysis, approximately 2.6 MWh of electrical energy are required in Europe including the energy loss by overpotential.[12,13] In this process, besides 1 t of Cl_2 also 1.127 t NaOH and 28 kg H_2 are formed, so the energy demand of 2.6 MWh relates to the production of all three materials. However, if the trichloride is used as indirect energy storage using renewable energy sources, the stored energy corresponds to the energy that would be required for a given amount of Cl_2.
1 t of $[NEt_3Me]Cl$ can store up to 0.79 t Cl_2 resulting in the corresponding trichloride $[NEt_3Me][Cl(Cl_2)_{1.68}]$.[6] The amount of chlorine per ton of the loaded storage ($[NEt_3Me][Cl(Cl_2)_{1.68}]$) can be calculated as

$$\frac{0.79\ \mathrm{t}}{1.79\ \mathrm{t}} = 0.44$$

Therefore, 1 t of $[NEt_3Me][Cl(Cl_2)_{1.68}]$ can indirectly store an energy of

$$0.44 \times 2.6\ \mathrm{MWh} = 1.1\ \mathrm{MWh}$$

If the chlorine production of two days of an industrial plant producing 1000 t Cl_2 per day would be stored, indirectly energy would be stored to an extend of:

$$1000\ \mathrm{t}\ \times 2 \times 2.6\ \frac{\mathrm{MWh}}{\mathrm{t}} = 5200\ \mathrm{MWh} = 5.2\ \mathrm{GWh}$$

If 2000 t of chlorine are stored as the Ionic Liquid $[NEt_3Me][Cl(Cl_2)_{1.68}]$, this storage would have a total mass of

$$\frac{2000\ \mathrm{t}}{0.44} = 4545\ \mathrm{t}$$

Considering the density of the Ionic Liquid ρ = 1.21 kg/L (at 25 °C and 1 atm),[6] the loaded Ionic Liquid would have a volume of

$$\frac{4545\ \mathrm{t}}{1.21\ \mathrm{t/m^3}} = 3760\ \mathrm{m^3}$$

As an Olympic swimming pool has a volume of 2500 m^3,[14] this corresponds to a volume of approximately 1.5 Olympic swimming pools.

Comparison to the pumped-storage plant Wehr

The pumped-storage plant Wehr has a water volume of V = 4,400,000 m^3 where the water has an average fall height of h = 625 m.[15] Given the density of water of ρ = 1000 kg/m^3 and the gravitation acceleration g = 9.81 m/s^2, the stored energy is:

$$E_{pot} = V \times \rho \times g \times h$$

$$E_{pot} = 4{,}400{,}000\ \mathrm{m^3} \times 1000 \frac{\mathrm{kg}}{\mathrm{m^3}} \times 9.81\ \frac{\mathrm{m}}{\mathrm{s^2}} \times 625\ \mathrm{m}$$

$$E_{pot} = 26{,}977{,}500{,}000{,}000\ \mathrm{J} = 7.5\ \mathrm{GWh}$$

Considering an efficiency of 80% (η = 0.8),[16] the usable energy is:

$$E_{use} = 7.5\ \mathrm{GWh}\ \times 0.8 = 6.0\ \mathrm{GWh}$$

References

[1] https://www.eurochlor.org/wp-content/uploads/2023/01/Industry-Review-2021-2022_FINAL-REVISED-2023-01-05.pdf (15.11.24).

[2] https://www.eurochlor.org/wp-content/uploads/2023/10/Chlor-Alkali-Industry-Review_CORRECTED-2023-10-06.pdf (15.11.24).

[3] https://www.statista.com/statistics/1002892/gross-electricity-production-in-europe/ (15.11.24).

[4] https://www.admin.ch/gov/en/start/documentation/media-releases.msg-id-94437.html (15.11.24).

[5] https://www.statista.com/statistics/550212/electricity-production-uk/ (15.11.24).

[6] P. Voßnacker, N. Schwarze, T. Keilhack, M. Kleoff, S. Steinhauer, Y. Schiesser, M. Paven, S. Yogendra, R. Weber, S. Riedel, *ACS Sustainable Chem. Eng.* **2022**, *10*, 9525–9531.

[7] https://www.intratec.us/products/primary-commodity-prices (15.11.24).

[8] https://www.imarcgroup.com/triethylamine-pricing-report (28.03.25).

[9] https://www.imarcgroup.com/chloromethane-pricing-report (28.03.25).

[10] W. N. Smith, J. McCloskey, **2002**, US6444846B1.

[11] M. S. Peters, K. D. Timmerhaus, Plant Design and Economics for Chemical Engineers, 4th ed., McGraw, **1991**, 197.

[12] https://www.eurochlor.org/wp-content/uploads/2018/06/12-Electrolysis-production-costs-November-2023.pdf (15.11.24).

[13] https://www.chlorineindustryreview.com/wp-content/uploads/2024/09/Chlor_Alkali_Industry_Review_2023_2024.pdf (30.10.24).

[14] https://www.merkur.de/lokales/ebersberg/arbeiten-fuer-neue-schule-in-vaterstetten-sind-ausgeschrieben-19-olympia-schwimmbecken-voller-erde-7190189.html (15.11.24).

[15] https://www.schluchseewerk.de/wp-content/uploads/2020/06/zm_technische-daten_wehr.pdf (15.11.24).

[16] https://www.verivox.de/strom/themen/pumpspeicherkraftwerk/ (15.11.24).
